# The Sleep-Immune Crosstalk in Health and Disease

**DOI:** 10.1152/physrev.00010.2018

**Published:** 2019-03-27

**Authors:** Luciana Besedovsky, Tanja Lange, Monika Haack

**Affiliations:** Institute of Medical Psychology and Behavioral Neurobiology, University of Tübingen, Tübingen, Germany; Department of Neurology, Beth Israel Deaconess Medical Center and Harvard Medical School, Boston, Massachusetts; and Department of Rheumatology and Clinical Immunology, University of Lübeck, Lübeck, Germany

## Abstract

Sleep and immunity are bidirectionally linked. Immune system activation alters sleep, and sleep in turn affects the innate and adaptive arm of our body’s defense system. Stimulation of the immune system by microbial challenges triggers an inflammatory response, which, depending on its magnitude and time course, can induce an increase in sleep duration and intensity, but also a disruption of sleep. Enhancement of sleep during an infection is assumed to feedback to the immune system to promote host defense. Indeed, sleep affects various immune parameters, is associated with a reduced infection risk, and can improve infection outcome and vaccination responses. The induction of a hormonal constellation that supports immune functions is one likely mechanism underlying the immune-supporting effects of sleep. In the absence of an infectious challenge, sleep appears to promote inflammatory homeostasis through effects on several inflammatory mediators, such as cytokines. This notion is supported by findings that prolonged sleep deficiency (e.g., short sleep duration, sleep disturbance) can lead to chronic, systemic low-grade inflammation and is associated with various diseases that have an inflammatory component, like diabetes, atherosclerosis, and neurodegeneration. Here, we review available data on this regulatory sleep-immune crosstalk, point out methodological challenges, and suggest questions open for future research.

## Graphical abstract

**Figure:**
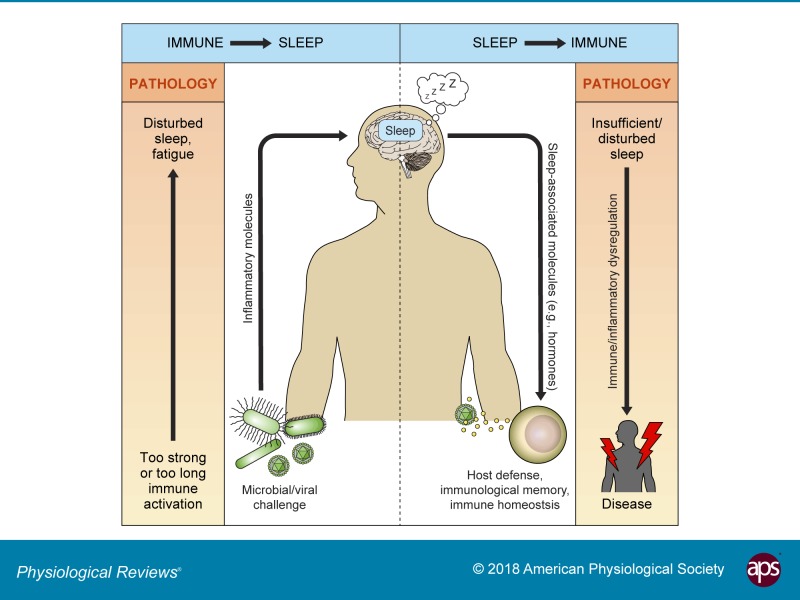

## I. INTRODUCTION

Sleep-immune interactions are well-known phenomena in everyday life and folk wisdom. There is no doubt that an infection makes us tired and increases the desire to sleep, and a good night’s sleep is commonly recommended as ‟the best medicineˮ for an infectious disease. Along this line, it is assumed that prolonged sleep loss weakens our body’s defense system and thus renders us more prone to catch a cold or any other infection. The scientific analyses of these notions started in 350 BC, when Artistotle elaborated in his book *On Sleep and Sleeplessness* that sleep is induced by hot vapors that arise from the stomach during digestion, and that a similar sleep response can be observed in feverish patients (16). In the early 20th century, researchers postulated a hypnotoxin that increases during wakefulness, induces sleep, and is cleared again during sleep (265, 333). The first hypnotoxin, discovered in the 1980s, turned out to be the bacterial cell wall component muramyl peptide, and like more than 2,000 yr ago, it was assumed that it derives from the gastrointestinal tract (304). By activating the immune system and the release of sleep regulatory substances like the cytokines tumor necrosis factor (TNF) and interleukin (IL)-1β, these muramyl peptides and other microbial products were shown in animal models to contribute to the homeostatic regulation of slow-wave sleep (SWS), the deepest form of sleep. We now know that both cytokines likewise mediate the SWS response to an infectious challenge (353). With respect to the sleep-to-immune directionality, early studies in the late 19th century showed that total sleep deprivation in dogs leads to death after several days (reviewed in Ref. 38). Later studies using more controlled approaches found that sleep deprivation of rats is lethal after ~2–3 wk (459), and a breakdown of host defense indicated by a systemic bacterial infection was reported after applying the same method of sleep deprivation (172, 175). Together with other experiments from recent times, these findings suggest an important role of sleep for immune defense (47, 79).

**FIGURE 1.**
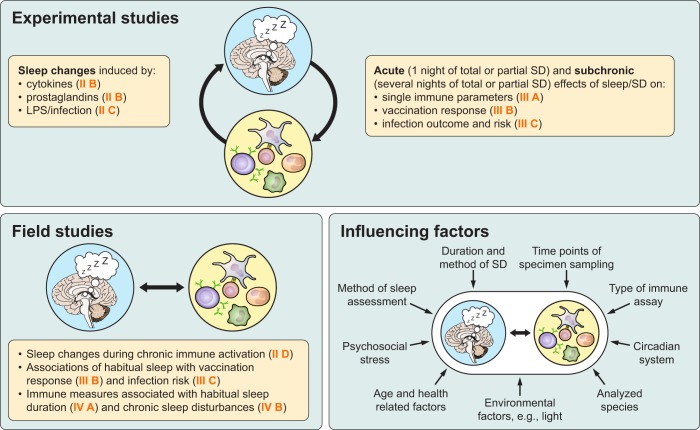
Research approaches for investigating sleep-immune interactions and potential influencing factors. Experimental studies investigate causal relationships between sleep and immune parameters by manipulating sleep or immunological factors, but have low ecologic validity (i.e., they cannot be easily translated into everyday situations). Field studies investigate naturally existing associations between sleep characteristics and immune parameters; however, causality cannot be inferred. Several factors (summarized in sect. VI*B*) influence the results of studies, including specifics of the study design, methods, and environmental factors. Orange numbers in brackets refer to the section dealing with the denoted topic. SD, sleep deprivation; LPS, lipopolysaccharide.

This review is based on findings from experimental, in-laboratory animal and human models that manipulate sleep or components of the immune system, as well as human field studies conducted in populations with various habitual sleep durations, chronic sleep disturbances, or chronic infectious or inflammatory diseases. It aims to summarize sleep changes in response to infectious and noninfectious challenges and to describe, on the other hand, the role of sleep in fine-tuning the immune system to foster immune defense. We start in section I by introducing basic aspects of sleep and the immune system and the means by which they can interact with each other. In section II, we outline how the components of the immune system signal to the brain and how sleep is altered during acute and chronic infectious or inflammatory diseases. Section III summarizes research that was performed mainly in the last two decades and assessed the sleep-to-immune directionality, including the impact of sleep on immune parameters and function, vaccination responses, and infection outcome and risk. This section includes mainly experimental studies in which sleep was actively manipulated. In section IV we focus on observational studies investigating the association between chronic sleep deficiency and immune parameters and describe how this association may contribute to increased disease risk. Section V summarizes data on studies examining possible countermeasures to reinstate immune balance, including recovery sleep following total or partial sleep deprivation, napping, extension of sleep duration, and cognitive behavioral therapy. Finally, in section VI, we provide conclusions on the available data, highlight the problems and pitfalls in this scientific field, and propose some questions open for future research. See FIGURE 1 for an overview of the topics covered by this review and the sections in which they are described.

### A. Sleep Characteristics, Regulation, and Measurement

Sleep is not a passive condition; the brain and the body are highly active during this particular behavioral state. Sleep can be characterized by a prototypical posture (usually recumbency), an increased arousal threshold with a reduced responsiveness to external stimuli, and a loss of consciousness. In contrast to coma, it can be easily reversed. Sleep is regulated homeostatically, meaning that sleep increases in duration and intensity after a prolonged period without sleep. In addition to this homeostatic component, a second process that is independent from prior wakefulness modulates the timing of sleep, namely, the circadian system. This system is responsible for imposing and synchronizing a close to 24-h rhythm on several behaviors and body functions, including the propensity to sleep or be awake along the 24-h sleep-wake cycle. The homeostatic and circadian components constitute the two factors of the so-called two-process model describing the regulation of sleep (64, 65). Several brain mechanisms are involved in this regulation, which have been reviewed in detail elsewhere (76).

**FIGURE 2.**
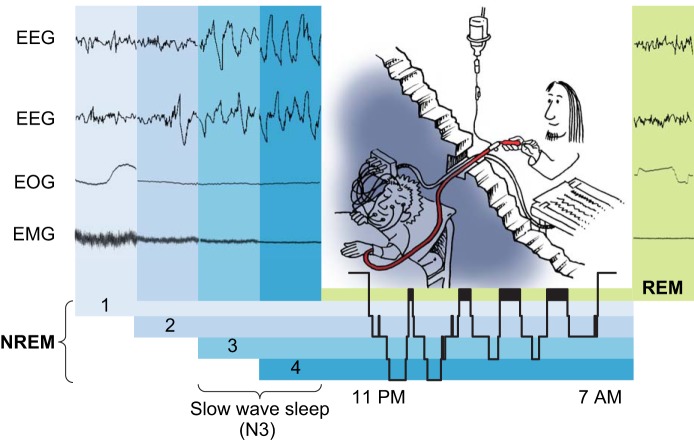
Prototypical hypnogram with EEG characteristics and sleep laboratory setting. Sleep is divided into stages N1, N2, N3 (formerly further subdivided into S3 and S4), and rapid-eye-movement (REM) sleep based on specific patterns of brain activity [measured with electroencephalography (EEG)], eye movements [measured with electroocculography (EOG)], and muscle activity [measured with electromyography (EMG)]. N1, N2, and N3 are collectively referred to as non-REM (NREM) sleep. The course of the different sleep stages across a sleep period is typically visualized in a hypnogram (*bottom right*). The typical sleep laboratory setting for measuring blood parameters during sleep includes a hole-through-the-wall system, allowing the blood sample to be collected from an adjacent room through a long tube without disturbing the participant’s sleep. (Adapted from Tanja Lange.)

The sleeping brain shows a characteristic pattern of activation, which can be measured by electroencephalography (EEG). Together with the measurements of eye movements (electrooculography, EOG) and muscle activity (electromyography, EMG), the EEG is used to discriminate between four stages of sleep (FIGURE 2). Sleep stage N1 refers to a transitionary state between sleep and wake and often includes slowly rolling eye movements. It is typically followed by sleep stage N2, which makes up ~50% of total sleep time in healthy young adults. N2 is characterized by the frequent occurrence of so-called sleep spindles (waxing and waning waves of 12–15 Hz) and K-complexes (single, high negative deflections) in the EEG. Around 20% of total sleep time is composed of sleep stage N3. It is distinguished from the other stages by a high incidence of low-frequency (≤4 Hz), high-amplitude (>75 µV) waves, called slow waves, and is therefore also referred to as SWS. Originally, this sleep stage was subdivided into two stages (S3 and S4), depending on the percentage of slow waves occurring (460), but this distinction has been abandoned in newer guidelines (8a). The fourth sleep stage is denoted rapid-eye-movement (REM) sleep due to the typical rapid eye movements that occur only during this sleep stage. Brain activity during REM sleep is very different from stages N2 and N3. It resembles more the waking brain activity, but also includes characteristic patterns like the so-called saw tooth waves. Most of the dreaming episodes seem to occur during REM sleep and muscle activity is actively suppressed, presumably to hinder acting out dreaming episodes. REM sleep makes up ~20% of total sleep time. Stages N1, N2, and N3 are collectively also termed non-REM (NREM) sleep as opposed to REM sleep. They typically occur in succession from light to deep sleep (i.e., from N1 to N3 with an increasing arousal threshold) followed by an episode of REM sleep. An average night with 8 h of sleep contains around five of these NREM-REM sleep cycles, which each last ~90 min (representing an ultradian rhythm). Whereas NREM sleep is predominantly under homeostatic control, REM sleep is mainly under circadian control. SWS predominates during the first half of the night and becomes reduced as sleep progresses. In contrast, REM sleep episodes are typically short at the beginning of the night and become longer and more frequent in the second sleep half.

Besides the different sleep stages, various other aspects of sleep can be determined, such as how fragmented the sleep period is (sleep fragmentation), how long a person needs to fall asleep (sleep onset latency), how long a person is asleep during the time in bed (sleep efficiency), and how much time a person spends awake after falling asleep until the final wake up (wake after sleep onset, WASO). All these features of sleep, which are interrelated, determine the quality of sleep. In addition, the density of the different EEG frequencies that characterize single sleep parameters like spindles and slow waves can be quantified by spectral analyses. Slow-wave activity (SWA), a measure of the density and amplitude of slow waves, is considered to reflect the intensity of sleep and is increased during sleep episodes that follow a prolonged period of wakefulness.

Sleep can be measured in several ways (TABLE 1). Polysomnography (PSG) is the gold standard for objectively measuring sleep in the laboratory. In addition to EEG, EMG, and EOG, PSG often includes measurements of heart rate, breathing functions, and blood oxygen saturation, especially in the clinical setting. Portable PSG devices allow the recording of sleep in the natural home environment. Actigraphy, the most common method for recording over periods of multiple days or weeks, employs an activity monitoring device (usually worn on the wrist) that estimates basic sleep parameters, such as sleep duration, sleep onset latency, and sleep fragmentation. To date, sleep stages cannot be differentiated using this method, and it is difficult to discriminate between sleep and immobility during wakefulness. However, actigraphy is practical for long-term recordings in the natural environment and provides an estimate of habitual sleep duration and day-to-day variability of various sleep parameters. A number of sleep parameters, such as sleep duration, sleep onset latency, WASO, number of nocturnal awakenings, as well as sleep quality (e.g., how refreshed do you feel this morning?) can also be assessed subjectively using diaries administered in electronic or paper form daily over a week or longer, or with standard questionnaires, such as the Pittsburgh Sleep Quality Index (PSQI) (87), which asks participants to report on their sleep habits in the past month. However, subjectively and objectively assessed sleep parameters can differ substantially (26, 327), which must be considered when evaluating sleep.

**Table 1.** Most common methods for measuring and experimentally manipulating sleep

***Methods for sleep measurement***	
Polysomnography (PSG)	Gold standard for objectively measuring sleep in the laboratory setting in humans. Consists of electrodes measuring brain activity (electroenephalography, EEG), eye movements (electrooculography, EOG), and muscle activity (electromyography, EMG). In the clinical setting, further measurements may include heart rate (electrocardiogram, ECG), respiratory events, snoring activity, blood oxygen saturation, and body position. Portable polysomnography (PSG) devices also exist for the ambulatory recordings in the home environment. While in humans the EEG electrodes are attached to the scalp, in animal experiments, the EEG electrodes are surgically implanted into the brain.
Actigraphy	Commonly used method to objectively estimate sleep parameters such as bedtimes, sleep duration, sleep onset latency, and sleep fragmentation over periods of multiple days in humans; it consists of a device that is typically worn on the wrist and that monitors motor activity (some models also measure light exposure and heart rate), based on which the various sleep parameters are estimated. It can be easily performed in the home environment.
Sleep diaries	Participants complete a sleep diary after awakening in the morning (and sometimes before going to bed to capture daytime functioning). These diaries allow subjective estimates of sleep duration, sleep onset latency, wake after sleep onset (WASO), number of awakenings, and sleep quality, among others.
Sleep questionnaires	Sleep questionnaires obtain information on sleep quantity and quality of the previous night or they can be filled in to retrospectively inform about average sleep quantity and quality and other sleep parameters over the last weeks.
***Methods for experimental sleep manipulations***	
Total sleep deprivation	Sleep is suppressed entirely for a certain period, typically for one entire night and the subsequent day in humans. Ideally, body posture, light exposure, and food and fluid consumption are controlled to resemble sleep conditions.
Partial sleep deprivation = sleep restriction	Sleep is suppressed only partly for a certain period, e.g., between 11 PM and 3 AM for one or several nights.
Sleep disruption	Sleep is interrupted repeatedly by forced awakenings of the participant or the animal for a limited time period (e.g., 30-min-long awakenings) and is used as a model for resembling sleep in, e.g., maintenance insomnia or parenting small children. Sleep can be also disrupted through frequent exposures to acoustic or mechanical stimuli, leading to frequent and brief EEG arousals (generally <10 s in length) and stage shifts into lighter sleep stages. This method is also referred to as sleep fragmentation and intends to model sleep that is characteristic for, e.g., breathing-related disorders or restless-leg syndrome.
Selective deprivation of single sleep stages	Slow-wave sleep (SWS) and rapid-eye movement (REM) sleep can be suppressed or reduced selectively. Selective SWS suppression is typically induced by delivering sounds to the sleeping participants as soon as the EEG shows certain features of SWS. The aim is to induce a shift into a lighter sleep stage without awakening the participant. Selective REM sleep suppression is performed more rarely in humans and participants typically have to be woken up if signs of REM sleep occur, as it is difficult to induce a shift to another sleep stage out of REM sleep. One of the most commonly used methods for long-term sleep deprivation in animal research, the multiple platform technique (see below), mainly suppresses REM sleep, while SWS is concomitantly reduced only to a certain extent.
Selective enhancement of single sleep stages	So far, a selective enhancement of single sleep stages is well established only for SWS. Certain aspects of SWS can be enhanced by delivering short sounds (e.g., pink noise) to sleeping participants, by applying weak electrical stimulation, or by using transcranial magnetic stimulation (TMS). These methods enhance or synchronize the occurrence of slow waves. Listening to specific hypnotic suggestions while falling asleep can increase the time spent in SWS.
Sleep extension	Sleep extension in humans can be performed by increasing the time allocated for the nighttime sleep period. Generally, time to go to bed will be advanced (e.g., from 11 PM to 10:30 PM) and time to get out of bed will be delayed (e.g., from 7 AM to 7:30 AM).
Daytime napping	Naps of different lengths (generally 30 min to ≤2 h) are scheduled during the daytime period to increase sleep duration across the 24-h day.
Disk-over-water method	Developed by Rechtschaffen and Bergmann in 1989 to induce sleep deprivation in rats. An experimental animal and a yoked control animal are housed on opposite sides of a divided disk suspended over water. Whenever the experimental animal falls asleep or reaches a specified sleep stage (as defined by EEG measures), the disk starts to rotate, forcing the animal to move to avoid falling into the water. The yoked control animal is allowed to sleep ad libitum, while experiencing the same amount of physical stimulation. This method can be used for total sleep deprivation or for deprivation of specific sleep stages, typically REM sleep.
Single platform technique	Originally developed for cats and later adapted for rats, this method involves the placement of the experimental animal on a small platform that is surrounded by water. Whenever REM sleep occurs, the animal partly or entirely falls into the water due to the muscle atonia occurring during REM sleep. Besides suppressing REM sleep, this method also suppresses SWS to a certain extent. The severe movement restriction, contact with water, and social isolation are factors that might induce a certain degree of stress in the exposed animals.
Multiple platform technique	Modification of the single platform technique including several platforms to limit the substantial movement restriction of the single platform technique. To reduce social isolation, in a modified version of this method, several animals are placed together in large tanks. This method has been also adapted for experiments in mice.
Gentle handling techniques	Animals are kept awake by receiving gentle physical stimulation by the experimenter (e.g., by gentle touching, cage tapping, cage movement, or exposure to novel objects). This method is typically used for shorter sleep deprivation protocols. It is suggested to be the least stressful method for depriving animals of sleep in the short term. However, longer periods of sleep deprivation are difficult to induce with this method because the handling by an experimenter is labor intensive and because it becomes increasingly difficult to keep animals awake over time.
Genetic modifications of sleep duration	Specific genetic modifications lead to a reduction or an increase of spontaneous sleep duration. This method is mainly used in *Drosophila* models.

### B. Cells, Mediators, and Tissues Involved in Host Defense

The immune system is our body’s defense system, which detects and eliminates internal and external threats. It is distributed throughout the body, and its cells, termed leukocytes or white blood cells (WBCs), are classified according to their ontogenetic origin, structural characteristics, surface markers, functions, and level of specificity. They are divided into two broad categories: *1*) cells of the unspecific, natural, innate immune system such as granulocytes (neutrophils, eosinophils, or basophils that develop into tissue mast cells), blood monocytes that develop into tissue macrophages [e.g., Langerhans cells in the skin, microglia-like macrophages in the brain (586)], dendritic cells (DCs), and unspecific lymphocytes such as natural killer (NK) cells; 2) cells of the specific, acquired, adaptive immune system, which either develop in the bone marrow (B lymphocytes or B cells) or in the thymus (T lymphocytes or T cells), and express unique receptors [B cell receptor, T cell receptor (TCR)] that recognize a specific antigenic peptide. Leukocytes originate, develop, and interact in primary (bone marrow, thymus) and secondary (lymph nodes, spleen, mucosa associated lymphatic tissue) lymphoid organs, and traffic among these lymphoid organs and tissues throughout the body via the bloodstream and lymphatic vessels. They communicate with each other and other non-immune cells via soluble mediators (cytokines, chemokines, shed surface molecules, vesicles, etc.) and direct cell-to-cell contact (involving surface molecules). Their defense mechanisms can be classified into humoral (soluble mediators) and cellular immunity (phagocytosis, cytotoxicity) and involve multiple escalation levels with increasing specificity.

At the body’s inner and outer surfaces, mucosal and skin epithelia are physical barriers protected by antimicrobial peptides, and complement factors that can also be produced by non-immune cells. If a pathogen nevertheless manages to invade the body by breaking these barriers, tissue resident macrophages or DCs recognize the challenge with pattern recognition receptors (PRRs), which are specialized innate immune sensors that are encoded in the germline. PRRs detect ‟non-selfˮ conserved pathogen-associated molecular patterns (PAMPs) from microbes like bacteria or viruses [e.g., bacterial lipopolysaccharide (LPS); viral double-stranded ribonucleic acid (RNA)] or endogenous danger/damage-associated molecular patterns (DAMPs), which are released by stressed or injured cells [e.g., heat-shock proteins (HSPs)] (51, 113, 368). PRRs in turn activate inflammatory signaling pathways like the inflammasome or NF-κB followed by the release of acute phase cytokines such as IL-1, IL-6, and TNF, of interferons (IFNs) with anti-viral activity, of vasoactive mediators like prostaglandins (PGs), and of chemokines that attract further leukocytes (chemotaxis). Locally, these processes result in the clinical signs of inflammation (redness, swelling, warming, pain, impaired function) and prompt innate defense mechanisms of phagocytosis or the release of antimicrobial substances that destroy the pathogen [e.g., reactive oxygen species (ROS)] (368, 378). Systemically, this acute phase response prompts central nervous symptoms like fever and sickness behavior (see sects. I*D* and II*A*).

**FIGURE 3.**
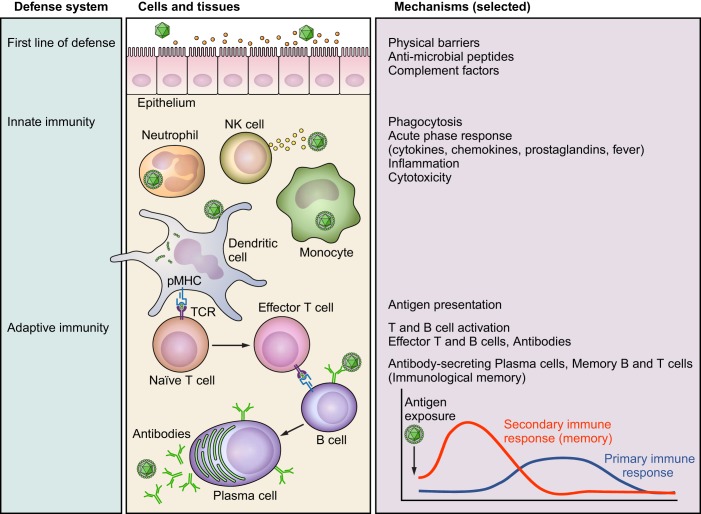
Major components of the immune system, a prototypical immune response to an infectious challenge, and immunological memory. The first line of defense against an infectious challenge are physical barriers and antimicrobial peptides. If a pathogen manages to pass these barriers, the innate immune system is activated. It includes phagocytotic and cytotoxic responses of leukocytes like neutrophils and natural killer cells (NK cells). If the pathogen cannot be cleared, the adaptive immune system becomes involved. Antigen-presenting cells (APCs, such as dendritic cells) present fragments of the pathogen (consisting of peptides) together with the major histocompatibility complex II (peptide-MHCII, pMHCII) to naive T cells. Those T cells with the matching T cell receptor (TCR) for the specific antigen then differentiate into effector T cells. Together with antigen-specific memory T and B cells, antigen-specific antibodies constitute the basis of immunological memory. Whereas the primary adaptive immune response to a pathogen takes several days to develop, the secondary (memory) response develops much faster and is more efficient (*bottom right*). The borders between the first line of defense, the innate immune response, and the adaptive immune response are fluid.

If the microbe evades this first line of defense, the adaptive immune system, consisting of T and B cells, can provide tailored, specific responses that are initiated in secondary lymphatic tissues but take several days to emerge. First, antigen-presenting cells (APCs) like DCs take up the antigen in tissues and migrate to secondary lymphoid organs like the lymph nodes. There, they present the antigenic peptide together with the major histocompatibility complex (MHC) II (peptide-MCHII, pMHCII) on their surface to naive CD4 T cells. This activates antigen-specific CD4 T cells (i.e., those cells with the matching TCR for the specific antigen), which then proliferate and differentiate into specialized effector CD4 T-helper (Th) cells (e.g., Th1, Th2, Th17). These cells in turn help macrophages, CD8 T cells, and B cells eliminate the pathogen. Cytotoxic CD8 T cells recognize pMHCI (e.g., on virus-infected cells) and kill the target cell by releasing cytotoxins (perforin, granzyme, granulosin). Activated B cells develop into plasma cells producing antibodies (immunoglobulins, Ig) that can specifically neutralize soluble antigens or mark cell-bound antigens for elimination. Depending on the type of infection and the pattern of PAMPs and DAMPs, APCs release certain cytokines, thereby determining CD4 T cell lineage commitment and eventually the class of antibodies [in humans, e.g., intracellular (viral and bacterial) infection: type I IFNs/IL-12, Th1, IgG1, IgG3; helminth infection: IL-4, Th2, IgG4, IgE; fungal infection: IL-23, Th17], hallmarking the close interplay between the innate and adaptive arm of the immune system (266, 368, 566). After elimination of the pathogen in the effector phase, most activated antigen-specific B and T cells die, but some persist as memory cells that allow a faster and more efficient response upon re-encounter of the antigen, and therefore represent immunological memory (181, 395, 577) (FIGURE 3).

### C. Low-Grade Inflammation

As mentioned in the previous section, the four cardinal signs of the typical inflammatory response are redness, swelling, heat, and pain (rubor et tumor cum calore et dolore). They were first described by Celsus in the 1st century. Much later, in the 19th century, Virchow added the fifth cardinal sign of inflammation, i.e., loss of function (function laesa), referring to the restricted function of inflamed tissue (reviewed in Ref. 354). The typical inflammatory response is triggered by a local infection or tissue injury, involving PAMPs and DAMPs, and is traditionally thought of as a protective reaction that defends and restores physiological functions (111, 296). The inflammatory mediators involved in this response are cytokines, chemokines, vasoactive amines, and PGs, which induce a variety of local biological processes (e.g., recruitment of neutrophils from the circulation to tissues at the site of infection/injury, vascular changes) with the goal of restoring homeostasis (378). In some cases, these inflammatory mediators may become detectable in the circulation and may act systemically (e.g., induce fever).

The function of restoring homeostasis can get lost if inflammation persists. In the past few decades, conditions characterized by mild systemic elevations of inflammatory markers have drastically increased, including obesity, type 2 diabetes, cardiovascular diseases, asthma, some chronic pain conditions, some forms of cancer, and neurodegenerative diseases. Furthermore, increases of inflammatory markers accompany the process of aging, a phenomenon termed inflammaging (96, 197). In these conditions, elevations of inflammatory markers are of low magnitude and the cardinal signs of local inflammation are generally absent. Such mild and often chronic elevations are most frequently referred to as low-grade inflammation; sometimes terms such as unresolved, sterile, meta-, sub-, or para-inflammation have been used. To assess low-grade inflammation, various measures usually associated with the innate immune system have been utilized, including C-reactive protein (CRP), IL-6, WBC counts, neutrophil counts, and platelet counts. The magnitude of the response may be defined as a two- to threefold elevation of such markers (297, 437). However, there is currently no consistent definition. Furthermore, the inducers of low-grade inflammation are not well understood. They do not appear to involve the classical inducers of the typical inflammatory response [i.e., infection or tissue injury (377)], but rather cellular stress or malfunction that leads to activation of PRRs by the release of DAMPs (e.g., HSPs) (68, 100). Additional inducers of low-grade inflammation are nutrients and metabolites acting as DAMPs (e.g., free fatty acids, oxidized lipoproteins) and commensal bacteria representing a source of PAMPs [sometimes also described with the more global term *microbe-associated molecular patterns* (MAMPs), as they are not necessarily pathogenic] (189, 240, 244, 295, 329, 347). Important ramifications of low-grade inflammation are insulin resistance, endothelial dysfunction, atherosclerosis, and neuroinflammation (109, 244, 336, 485, 572), as well as immunodeficiency due to impaired DC, T, and B cell functions (80, 199, 422), which in turn can promote failure of tumor and pathogen defense (123, 426) (reviewed in Ref. 198). As will be summarized in section IV, sleep deficiency, either in the form of short sleep duration or sleep disturbance, appears to be one behavioral trigger of low-grade inflammation and associated diseases.

### D. Basic Mechanisms of Neuro-immune Interactions

The central nervous system (CNS) and the immune system are the two super-systems that can sense environmental stimuli, generate appropriate responses, and commit this knowledge to memory such that the organism is prepared for stimulus re-encounter and continuously adapts to its environment. In performing these tasks, the two systems closely interact: an acute mental or physical stressor that primarily activates systems under CNS control, i.e., the neuroendocrine hypothalamus-pituitary-adrenal (HPA) axis and the autonomic nervous system (ANS), will also induce an inflammatory response (52, 525). On the other hand, a microbial challenge that primarily activates the immune system will also prompt neurobehavioral, neuroendocrine, and ANS responses (42, 132). A reasonable interpretation of the first phenomenon is the idea that in ancestral times any kind of stressor (e.g., facing a predator or a conspecific enemy) also challenged the body’s integrity and that the immediate activation of the immune system along with the freeze, fight, or flight response (which is initiated by the ANS) helped to ward off pathogens invading the wound and to initiate the healing process (383). The second phenomenon reflects the concept of the immune system being our sixth sense that recognizes stimuli that we otherwise cannot see, hear, taste, touch, or smell and signals this information to the brain (56, 58). To optimize ensuing defense mechanisms, the CNS in turn generates a set of measurable changes in body functions and behavior, such as fever, activation of neuroendocrine axes and the ANS, as well as sickness behavior, which is characterized by inactivity, fatigue, sleep alterations, anhedonia, reduced responsiveness to external stimuli, social withdrawal, anorexia, adipsia, and increased pain sensitivity (130, 372).

Research in the interdisciplinary field of (psycho)neuroimmunology elucidated anatomic and molecular mechanisms underlying these bidirectional brain-immune interactions that were summarized in comprehensive reviews in previous issues of *Physiological Reviews* (57, 129, 350, 549). Therefore, we will only briefly outline basic principles of this crosstalk. The CNS and the immune system are linked by nerve fibers, soluble mediators, and leukocyte traffic to the brain and the spinal cord. Primary and secondary lymphoid organs are innervated by sympathetic, peptidergic, and sensory nerve fibers (182). Numerous neurotransmitters and neuropeptides of efferent nerves (like catecholamines or neuropeptide Y) can be recognized by matching receptors on immune cells, and leukocytes themselves can synthesize and release these neuronal messengers (e.g., Refs. 190, 414, 436). The cholinergic innervation of immune organs is still a matter of debate (206, 400), yet a non-neuronal cholinergic system in T cells can locally provide acetylcholine release in the periphery (99, 203). Apart from lymphoid organs, tissue-resident leukocytes like macrophages can directly sense neurotransmitters and neuropeptides that are released by local nerve endings (414). In contrast to this fast communication pathway through nerve fibers, the neuroendocrine axes provide an additional, somewhat slower control of the immune system. Leukocytes can sense and synthesize a plethora of neuroendocrine mediators of the hypothalamic, pituitary, and glandular level [e.g., corticotropin releasing hormone, adrenocorticotropic hormone, cortisol; growth hormone releasing hormone, growth hormone (GH), insulin-like growth factor-1; dopamine, prolactin] and can even form an own, local neuroendocrine axis in an autocrine and paracrine manner (e.g., Refs. 39, 155, 401, 414, 576).

The capability of leukocytes to produce neurotransmitters, neuropeptides, and hormones allows them to signal to the brain via afferent nerve fibers and the bloodstream. Further mediators of immune-to-brain signaling are immunopeptides including cytokines and chemokines that are produced by immune cells and act on neurons, astrocytes, and microglia of the peripheral nervous system (PNS) or the CNS via respective receptors. These chemicals and other inflammatory agents like PGs, PAMPs, and DAMPs can reach the PNS or CNS by crossing the blood-brain barrier via transporter systems or at leaky sites of circumventricular organs. Alternative routes of immune-to-brain signaling are immunomediators that can activate vagal and other neural afferents, as well as cerebral endothelial and perivascular cells or the directed traffic of leukocytes to the brain (70, 503, 523, 586). All these pathways may induce microglial activation and neuroinflammation in response to peripheral inflammation, thereby initiating CNS responses including fever and sickness behavior (486, 493). Autoreactive T and B cells add an additional level of complexity, as they can regulate inflammatory processes in the brain (291, 499) and might represent a reflection of ‟our self,ˮ the ‟immunculus,ˮ to restore homeostasis of tissues and organs (441). Neurons, glia cells, and nerve fibers themselves can synthesize cytokines and chemokines, demonstrating a further pathway of brain-to-immune signaling (70, 592). In sum, the outlined results show that there is a common biochemical language for intra- and intersystem communication between the CNS and the immune system, and the classification into *neuro*transmitters, *neuro*peptides, hormones, and *immuno*mediators is based on the chronology of their discovery rather than on the unique assignment to a system.

The final outcomes of these brain-to-immune or immune-to-brain signaling pathways are determined by the specific experimental setup employed to measure the neuro-immune interactions of interest. For example, activation of the classical HPA and ANS stress axes can support or suppress inflammatory and adaptive immune responses in human and animal models, depending, among other factors, on whether it occurs before or after the immunological challenge (274, 371, 474). Some neurotransmitters, neuropeptides, and hormones can therefore not be classified in general as either immunosuppressive or immunosupportive, but rather act in one or the other way depending on the specific situation. The general picture suggests that the bidirectional brain-immune crosstalk serves to fine-tune the immune response that is normally characterized by four Rs: recognition, response, regulation, and resolution. Recognition and response take place during the early immediate pro-inflammatory phase that is supported by actions of the stress axes, such as priming of immune cells and inflammation, and mobilization of energy substrates to fuel the immune system (86, 288, 528). Regulation and resolution refer to subsequent counter-inflammatory measures that limit the inflammatory process in time and space. During this phase, the immune system has two levels of regulation. At one level, the immune system monitors itself by various intrinsic control mechanisms and pro-resolving mediators [e.g., regulatory T cells (Treg), indoleamine 2,3-dioxygenase, M2 macrophages, IL-10, resolution-associated molecular patterns (RAMPs), and specialized pro-resolving lipid mediators] (381, 483, 501, 507, 510). At the other level, the CNS exerts a superordinate extrinsic control through its efferent pathways (i.e., HPA and ANS stress axes) (44, 164). This balanced and coordinated control ensures a highly efficient, short-lasting, local immune response at low energy costs and with minimal collateral damage (139, 526).

## II. THE SLEEP RESPONSE TO IMMUNE ACTIVATION

### A. Fever and Sickness Behavior

As outlined above, acute infectious illnesses cause CNS responses such as fever (230) and a set of common symptoms of sickness, including fatigue, sleepiness, social withdrawal, negative mood (depression, anxiety), pain hypersensitivity, and decreased appetite (131). These changes are considered adaptive responses of the CNS and presumably aid the recovery from infections. For example, fatigue and sleepiness promote a less active behavioral state, thereby likely facilitating recovery from infection by conserving energy (560). Over the last 30 yr, it has been well established that fever and sickness behavior in response to infection or inflammatory diseases are mediated by inflammatory mediators, including cytokines and PGs that signal to the brain (131, 487). In the following, the role of these mediators in sleep modulation will be reviewed in the context of spontaneous, physiological sleep (sect. II*B*), acute immune activation following infectious or inflammatory challenges (sect. II*C*), and chronic immune activation related to infectious and inflammatory diseases (sect. II*D*).

### B. Inflammatory Mediators Involved in Physiological Sleep Regulation

#### 1. Cytokines

The first discovery suggesting the existence of a central sleep-promoting substance dates back to 1909, when Ishimori in Japan and Piéron in France reported that sleep in well-rested dogs can be induced by injecting cerebrospinal fluid (CSF) from sleep-deprived dogs (265, 333). These early findings were supported by numerous follow-up studies using EEG recordings to objectively quantify sleep in various animal species (66). In parallel, scientists were searching for a sleep-promoting substance in brain and body fluids. In 1975, Pappenheimer finally extracted a substance from the CSF and brain tissue of sleep-deprived animals, which he called ‟factor Sˮ (S stands for sleep promoting) (424). Shortly afterwards, this factor was identified as a muramyl peptide (304), which is produced during phagocytosis of bacteria by macrophages (272). At that time, it was already known that muramyl peptides induce the production of cytokines by macrophages (332). Over the following years, the role of cytokines (in particular IL-1 and TNF) in homeostatic NREM sleep regulation has been intensively studied in animals. To be considered a sleep regulatory substance, administration of the substance should increase sleep amount, whereas inhibiting the biological action or production should result in a decrease of spontaneous sleep, and the diurnal variations in the endogenous synthesis should parallel sleep-wake behavior (118). To investigate sleep-regulatory properties of cytokines, cytokine antagonists [e.g., IL-1 receptor antagonist (IL-1ra) or anti-IL-1 antibody] have been used to prevent their biological actions. To summarize the findings, blocking the biological actions of the cytokines IL-1 and TNF resulted in a reduction of physiological NREM sleep amount or NREM sleep rebound after sleep deprivation. On the other hand, increasing the availability of those cytokines promoted NREM sleep amount and intensity and suppressed REM sleep amount. These findings established both cytokines, IL-1 and TNF, as substances involved in the homeostatic regulation of sleep (for review, see Refs. 299, 411). Of note, increased and intensified NREM and decreased REM sleep depend on several factors, such as time of day, route, and dose of administration. For example, in rats, NREM sleep-increasing effects of IL-1 occur only within a small dose-window: lower doses of IL-1 increase NREM sleep without inducing fever; higher doses of IL-1 accompanied by fever increase and fragment NREM but decrease REM sleep; and even higher doses decrease both (412) (as reviewed in Ref. 300).

Other cytokines, including IFN, IL-2, IL-4, IL-6, IL-10, IL-13, IL-15, and IL-18 also appear to have some sleep regulatory properties. The anti-inflammatory cytokines IL-4, IL-10, and IL-13 have been reported to attenuate NREM sleep amount in rabbits (308, 314, 315), while the pro-inflammatory acting cytokines IFN-γ, IL-2, IL-6, IL-15, and IL-18 have NREM sleep-promoting actions in animal models (238, 306, 307, 309). However, these cytokines have received much less attention than IL-1 or TNF (411). Thus their role in physiological sleep regulation is much less clear. Overall, animal studies suggest that most pro-inflammatory cytokines are NREM sleep promoting, while anti-inflammatory cytokines are NREM sleep reducing.

In humans, only very few studies have investigated the involvement of cytokines in the regulation of physiological sleep. Circulating levels of IL-1, TNF, and IL-6 have often been found to peak during sleep or in the early morning hours (reviewed in Refs. 95, 321). Such findings may suggest the involvement of these cytokines in the regulation of physiological sleep-wake behavior. Studies comparing the sleep period against a wake period at night clarify to what degree such diurnal variations are sleep-wake dependent or result from a circadian, sleep-independent process (see sect. III). While there is clear evidence that administration of cytokines (in particular IFNs and IL-2) to patients suffering from various forms of cancer, multiple sclerosis, rheumatoid arthritis (RA), human immunodeficiency virus (HIV), or hepatitis may result in increased subjective sleepiness and other sickness behavior symptoms, changes in objectively assessed sleep in these patients have not been studied (4, 382). Administration of IFN-α (520) or IL-2 (324) in healthy participants did not change PSG-derived sleep parameters the following night. With respect to the cytokine IL-6, subcutaneous administration before sleep initiation in healthy participants resulted in a delayed REM sleep latency and reduced REM sleep amount, without a change in total NREM sleep amount (519). Intranasal administration of IL-6 in healthy participants, however, intensified SWS in the second half of the night (increased SWA), although no overall changes in the amount of NREM sleep were observed (36). A reduction of SWS amount and intensity has been observed in the beginning of the night following administration of granulocyte colony-stimulating factor, which in parallel led to an increase of the plasma levels of endogenous antagonists of IL-1 and TNF, i.e., IL-1ra and soluble TNF receptors, respectively (497). These findings suggest the involvement of IL-1 and TNF in the physiological regulation of sleep in humans. However, two recent studies in healthy participants revealed an increase, rather than the expected decrease, in SWA following administration of the anti-inflammatory drugs anakinra, an IL-1ra (495), and minocycline (50), a tetracycline that among many other actions suppresses central nervous TNF production (210). These human findings contrast with studies in animals showing that pro-inflammatory, rather than anti-inflammatory, activity enhances sleep. Such discrepancies highlight the need to further investigate the role of pro- and anti-inflammatory activities in human models, as their sleep modulatory effects may differ from those observed in animal models.

#### 2. Prostaglandins

Sleep regulatory effects have also been reported for PGs. PGs are lipid mediators that are synthesized de novo from the omega-6 fatty acid arachidonic acid through actions of cyclooxygenase (COX)-1 and COX-2 enzymes and specific synthases. PGs mediate some of the cardinal symptoms of inflammation, such as fever and pain. Their involvement in the production of such symptoms is demonstrated by the therapeutic effects of nonsteroidal anti-inflammatory drugs (NSAIDs), e.g., ibuprofen or acetylsalicylic acid (aspirin), which primarily prevent the synthesis of PGs through inhibition of COX enzymes (558). More recently, the PG system has also been shown to play a critical role in the resolution of inflammation (502, 531) and therefore may also contribute to unresolved or chronic low-grade inflammation if dysregulated.

In the 1980s, work by Hayaishi and colleagues identified PGD_2_, the most abundant prostanoid in the brain of rodents, as a potent, sleep-promoting substance in rats and mice (reviewed in Refs. 246, 551). Infusion of PGD_2_ into the intracerebroventricular (ICV) (particularly the subarachnoid) space in rats induced substantial increases of NREM sleep (252, 366). Inhibition of PG production by COX-2 inhibitors reduced spontaneous and TNF-induced increases in NREM sleep in the animal model (538, 595), supporting the role of PGs in sleep modulation. Furthermore, moderate sleep deprivation caused an increase in CSF levels of PGD_2_, E_2_, and F_2α_ in rats (455). Such upregulation of various PGs suggests that sleep loss may target COX enzymes, the precursor of all functional PGs, rather than a specific PG.

In humans, PGs have rarely been studied in the context of sleep. This may relate to difficulties in the reliable assessment of PGs in the blood, given the short half-life and rapid metabolization of PGs (498). However, precursors (e.g., synthases) or metabolic products of PG appear to be more biologically stable and therefore provide a reliable estimate of PG production. In healthy participants, serum concentrations of lipocalin-type PGD synthase, which catalyzes the conversion from PGH_2_ to PGD_2_, have been reported to vary throughout the day, with highest levels at night (sleep period) and lowest levels in the afternoon. Total sleep deprivation blunted the nocturnal increase at night and elevated levels during the daytime (275), suggesting a role of PGD_2_ in human sleep physiology. With the use of a model of prolonged experimental sleep restriction of 10 days, a nonsignificant increase of PGD_2_ and PGE_2_ metabolites of ~20% has been reported in healthy participants (223), while total sleep deprivation of 3 days produced increases of PGE_2_ of almost 30% when compared with control sleepers (221). A few studies have also addressed whether inhibition of PG production through NSAIDs affected sleep. Indeed, acute administration of aspirin at the recommended daily dose range in healthy participants disrupted sleep (i.e., decreased sleep efficiency, increased number of awakenings) (398) and decreased SWS (241). Acute administration of ibuprofen, in addition to its sleep disrupting effect, also led to a delay of SWS (398). While these findings suggest the involvement of the PG system in human sleep physiology, the exact mechanisms and PGs involved remain unknown. The effects of chronic administration of NSAIDs on sleep are also unknown. Given that a large proportion of the population uses NSAIDs on a regular basis (135), future research may address the long-term effects on sleep physiology. In addition, in light of more recent findings that the PG system not only promotes inflammation, but also plays an essential role in resolving inflammation (502), blocking this system through NSAIDs may contribute to ongoing, unresolved inflammation in response to sleep deficiency. Thus further investigations of the PG system may be of critical importance in elucidating mechanisms underlying the association between sleep deficiency and low-grade inflammation in humans, which is described in section IV.

#### 3. Upstream signals of sleep regulatory substances

**FIGURE 4.**
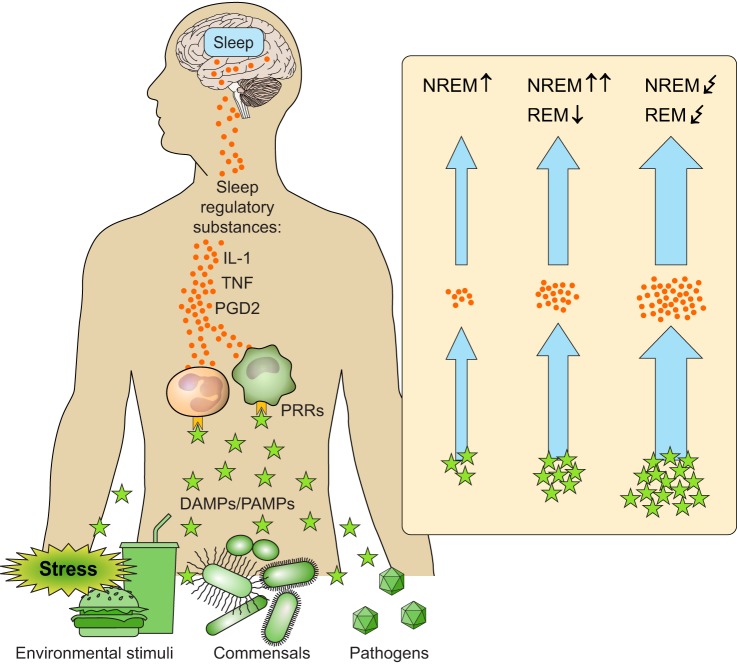
Conceptual model of sleep changes in response to immune activation and underlying mechanisms. Environmental stimuli (e.g., food intake, stress), commensal bacteria, and infectious pathogens (here illustrated as viruses) are recognized by the immune system as damage- and pathogen-associated molecular patterns (DAMPs and PAMPs, green stars), which activate pattern recognition receptors (PRRs, orange polygon) on innate leukocytes. This PRR activation induces an inflammatory response with the production of sleep regulatory substances, such as interleukin (IL)-1 and tumor necrosis factor (TNF) (both represented by orange dots), which reach the brain and promote non-rapid-eye-movement (NREM) sleep (left arrow). In higher doses (e.g., during an infection; middle arrow), these sleep regulatory substances may also suppress rapid-eye-movement (REM) sleep. Prostaglandin (PG) D_2_ is shown as a potential further mediator of sleep changes in response to immune activation. These sleep responses to immune activation are assumed to be adaptive. Subtle immune activation may be involved in homeostatic NREM sleep regulation that in turn could serve to restore immune homeostasis. More pronounced immune activation during an infection can induce a sleep response that in turn may support host defense and immunological memory formation. However, an extreme immune activation (e.g., during severe infection; right arrow) seems to disrupt both NREM and REM sleep, often accompanied by sleep fragmentation, feelings of nonrestorative sleep, and daytime fatigue. Notably, most of our knowledge is based on animal research, and confirmation in humans is still needed.

A muramyl peptide was identified as the upstream signal of sleep regulatory substances, in particular IL-1 and TNF (304). Although it is still unknown why muramyl peptides increase during sleep deprivation, it was assumed that commensal gut bacteria contribute to their systemic release (304). This notion is supported by animal studies showing reduced NREM sleep in rats treated with antibiotics targeting gut bacteria (74) and of altered sleep in rats and mice following the dietary supplementation of commensals (385, 542). Furthermore, recent data support a key role of gut microbiota and corresponding microbiota-derived PAMPs in central nervous processes like mood, cognition, pain, and eating behavior in animals, and there is also first evidence for such a relationship in humans (85, 150, 151, 394). It is tempting to speculate that increases in systemic levels of microbiota-derived PAMPs and inflammatory mediators following food intake likewise mediate post-prandial sleep changes (415, 599). Apart from microbiota-derived PAMPs, low levels of PAMPs that are recognized during the daily, asymptomatic confrontation of our immune system with numerous infectious bacteria, viruses, or fungi could trigger subtle increases in systemic cytokine and PG levels. Finally, DAMPs are released into the extracellular space not only during cell damage, but also during regular cellular activity. For example, they increase in the brain during synaptic firing (305) and in the periphery during acute mental (52, 273) or physical stress (13). Furthermore, in humans, DAMPs increase in the brain (509), CSF (410), and blood (35) following acute sleep deprivation. Conceivably any interaction of an organism with the environment, including learning, stress, social contacts, physical activity, and food intake, prompts the release of PAMPs and/or DAMPs (FIGURE 4). They can activate the immune system through binding to respective PRRs, which likely leads to systemic increases of sleep regulatory substances, such as TNF and IL-1 (303, 305). These substances appear to keep track of the organisms’ prior activity and, upon reaching a certain threshold, contribute to the induction of sleepiness and eventually NREM sleep. This view may help to explain findings of inflammatory responses (18) and sleep changes, such as an increase in NREM sleep amount (2, 555) observed in laboratory animals housed in enriched or novel environments. However, the causal link between immune and sleep responses in the context of enriched environments still needs to be established in future research. The involvement of PRRs in this ‟use-dependentˮ NREM sleep induction (305) is supported by findings in mice devoid of two important classes of PRR: the Toll-like receptors (TLRs) (585) and the nucleotide-binding oligomerization domain-like receptors (Nod-like receptors, NLRs) (605).

Collectively, there is ample evidence that IL-1 and TNF, in particular, and PGD_2_, play a critical role in the regulation of physiological, spontaneous NREM sleep in animals, and potentially in humans, while they may not be involved in physiological REM sleep regulation (250, 551). Other substances, such as IL-6, have received much less attention, and their role in sleep regulation is therefore less understood. PAMPs, DAMPs, and respective PRRs may form a mechanism underlying the release of sleep-regulatory substances; however, further investigations are warranted on this topic. See FIGURE 4 for a conceptual summary. During an infectious challenge, increases in NREM sleep amount or intensity (i.e., increased SWA) and decreases in REM sleep amount (353) have been observed. As outlined in the next section, the release of IL-1 and TNF appear to be key mediators of this effect. It is worth mentioning that a number of other factors are involved in the regulation of sleep and may interact with the immune system to promote or inhibit sleep. These include, among others, hormones of the HPA and the somatotropic axes; neurotransmitters such as acetylcholine, serotonin, norepinephrine, histamine, and dopamine; neuropeptides such as orexin; the nucleoside adenosine; and the hormone melatonin. The interested reader is referred to Reference 76 for a comprehensive review on sleep regulation.

### C. Sleep Response to Acute Immune Activation Following an Infectious or Inflammatory Challenge

One-hundred years ago, during World War I, the neurologist Constantin Freiherr von Economo described for the first time the disease ‟encephalitis lethargicaˮ (568), which spread across the world from ~1916 into the 1930s and was frequently characterized by pathologically increased sleep. Von Economo initially assumed that an infectious virus and related inflammatory processes were the cause of Encephalitis Lethargica. This was supported by findings showing that the disease could be transmitted via brain tissue from a deceased patient to a monkey (569). Although the majority of recent findings deputed the infectious disease hypothesis (reviewed in Ref. 40), the idea that an infection per se can alter sleep behavior found support in later studies in humans and animals.

Starting in the 1980s, the sleep response to infectious challenges has been intensively studied in animal models, including rats, rabbits, mice, and, more recently, flies. Substantial evidence has accumulated over the last decades supporting the view that pathogens, including bacteria, viruses, fungi, and parasites increase the amount and intensity of NREM sleep while decreasing the amount of REM sleep (for comprehensive review, see Refs. 299, 353). Of note, pathogen-induced effects on NREM and REM sleep amount and intensity are complex and depend on the pathogen structure, the dose administered, and time of day of administration. In addition, a biphasic time course of changes in NREM and REM sleep amount has been reported after inoculation with bacteria (*Staphylococcus aureus*) in rabbits (544) and bacterial cell wall components (endotoxin) in rats (318), such that the initial increase in NREM and suppression in REM sleep were reversed at later time points. Detection of such a biphasic pattern of NREM and REM sleep require long EEG recording times (e.g., up to 48 h) and suggest a counterregulatory response to the initial sleep changes induced by pathogens.

The mediators underlying this sleep-modulating effect are components and/or decomposition products of these pathogens, including PAMPs such as endotoxins (e.g., LPS), lipid A, muramyl peptides, viral double-stranded RNA, respective PRRs like TLRs and NLRs, as well as downstream pathways like the transcription factor NF-κB and inflammasomes, all of which are known to induce cytokines and PGs (301, 352, 605). As reviewed in section II*B*, IL-1 and TNF are key mediators of sleep-wake alterations and exert their NREM sleep-enhancing and intensifying effects also during an infectious challenge. Using knockout mice for various PG receptors and the COX inhibitor meloxicam, a recent study concluded that the LPS-mediated increases in NREM sleep are only partially dependent on PGs (407), corroborating the view that the sleep response to an infection is mainly triggered by IL-1 and TNF. Given that increases in core body temperature can promote NREM sleep (488), it is important to note that the infection-driven NREM sleep response is independent from the fever response (303).

Due to ethical reasons, there are only a few research studies where live pathogens have been administered to humans. Smith and colleagues found in a series of experiments in the late 1980s that experimentally induced influenza A and B and rhinovirus-induced common colds in healthy participants led to an increase of self-reported sleep duration in the symptomatic phase of the illness (517). Interestingly, changes in self-reported sleep duration were also evident in the incubation phase of the illness, before symptom development (for review, see Ref. 517). In the only study that, to our knowledge, recorded sleep using PSG in this context, rhinovirus-type-23-induced common colds disrupted sleep in symptomatic individuals, as evidenced by reduced total sleep time and reduced sleep efficiency in the active phase of the illness (158).

Most human studies investigating the role of sleep in host defense mechanisms have employed experimental models of acute infections with well-characterized host responses, such as those induced by vaccines and bacterial endotoxins. While most vaccination studies have examined the effects of naturally occurring or experimentally induced sleep loss on immunological responses (reviewed in sect. III*B*), studies investigating alterations of sleep itself in response to vaccination are rare. *Salmonella typhi* (typhoid) vaccination administered in the late afternoon in healthy participants led to a mild inflammatory response with elevated IL-6 levels 2 h post-vaccination, and while well tolerated, PSG assessed sleep in the night following vaccination was characterized by sleep disturbances (e.g., increased number of nighttime awakenings) compared with those who received a placebo injection (505). Vaccinations with hepatitis A virus (320, 325) and influenza A H1N1 (swine flu) virus (34) in healthy participants appeared to not cause any gross sleep changes in the night following the vaccination, but there was no comparison of sleep data to a placebo-vaccinated control group in these studies. However, specific sleep parameters, such as SWS intensity, were predictive of the magnitude of the antibody response (320). Thus intensity of SWS may play a critical role in the optimal formation of an antigen-specific immune response (see sect. III*B*). In the 1990s, Pollmächer and colleagues performed a series of experiments on the effects of low-dose endotoxin challenge on sleep in humans (e.g., Refs. 224, 396, 442). Endotoxins are the major cell wall component of gram-negative bacteria and important PAMPs that activate the innate immune system during the initial phase of bacterial infection through TLRs. When injected intravenously, endotoxin induces a host response that includes increases in TNF and TNF receptors within the first hour of injection; increases in IL-6, IL-10, IL-1ra, and other cytokines; and increases in leukocytes, GH, cortisol, body temperature, and heart rate. It also dose-dependently leads to flulike sickness symptoms such as headache, muscle pain, and nausea. These endotoxin-induced responses return to baseline after 8 to 12 h (for comprehensive reviews, see Refs. 443, 493). Collectively, these studies have shown that in humans, NREM sleep enhancing or intensifying effects are restricted to times of very mild host response activation, whereas stronger responses characterized by large cytokine increases and fever caused sleep disruption (224, 396, 442).

In summary, findings in both animals and humans show that pathogens or their components are able to alter sleep. Observations of enhanced or intensified sleep in response to acute infectious challenges have led to the hypothesis that sleep is beneficial for host defense mechanisms and/or host recovery. Findings by Toth et al. (546) support this hypothesis. They reported that rabbits inoculated with *Escherichia coli* bacteria had less morbidity and mortality when NREM sleep was enhanced in response to the infectious challenge. A potential explanation for this immune-supportive effect is that sleep may facilitate energy allocation to the immune system (496). In contrast to the infection-driven increase in NREM sleep duration and intensity, animal and human studies have repeatedly shown a concomitant suppression of REM sleep. As REM sleep is incompatible with thermoregulatory effector responses like shivering, REM sleep suppression may promote the generation of fever that in turn boosts immunity (250). In humans, the hypothesis of a beneficial effect of sleep on host defense mechanisms has been mostly studied in the context of sleep loss, either naturally occurring or experimentally induced. These studies, together with respective animal experiments, will be reviewed in section III, with a major focus on studies using experimental modulation of sleep.

### D. Sleep Changes During Chronic Immune Activation Related to Infectious or Inflammatory Diseases

While most investigations in this area have reported on prevalence rates of sleep disturbances or focused on the characterization of the sleep-wake profile, studies specifically linking observed sleep changes to immunopathology are still rare. In the following, a number of chronic infectious and inflammatory diseases will be reviewed to exemplify the variety of self-reported and objective sleep changes observed among different diseases, and to report on the relationship between sleep changes and immunopathology. We here mainly focus on human diseases, as the relationship between sleep and immunopathology has been less frequently addressed in animal models, and for some chronic infectious or inflammatory diseases, animal models do not exist, such as for myalgic encephalomyelitis/chronic fatigue syndrome (ME/CFS).

#### 1. Chronic infectious diseases

With respect to HIV infection, two recent systematic reviews revealed a prevalence rate of self-reported sleep disturbances of ~60% in people living with HIV, which is much higher than the prevalence rate of 10% in the general population (97, 591). Based on objective polysomnographic assessment, sleep structure changes existed during the early asymptomatic stage of the disease, characterized by a robust increase in SWS in particular during the latter part of the sleep period. In the advanced stages of HIV infection, typical PSG findings were sleep disruption with frequent nighttime awakenings and arousals, resulting in low sleep efficiency (reviewed in Ref. 134). Inflammatory cytokines (e.g., IL-1 and TNF) appear to be likely candidates in mediating sleep changes observed in HIV infection, given their sleep modulating role in animal models (see sect. II*B1*) and upregulation in HIV-infected patients (195, 477, 540). Indeed, some of the very few studies conducted in this area reported an association between sleep parameters (i.e., SWA, sleep duration) and pro-inflammatory cytokine dysregulation in HIV-infected individuals (133, 195). Such associations appear to be influenced by disease severity and duration. For example, the correlation between TNF and SWA has been shown to weaken as a function of days since seroconversion (133). Furthermore, in a recent study performed in low-income ethnic minority women living with HIV, greater self-reported sleep disturbances were related to lower CD4 T-cell counts. This relationship persisted after adjusting for the effects of mood, stress, and disease status, suggesting that the relationship was independent of such factors (500). Of note, even after the initiation of antiretroviral therapy, HIV-positive patients continue to report lower sleep quality (359, 539, 591), and in a recent study in a South African-treated HIV-positive population, an increase in CD4 T-cell counts was associated with lower sleep quality (461). In animals, injection of the HIV envelope glycoprotein 120 in the brain of rats caused an increase of hypothalamic mRNA expression of IL-1 and IL-10 and an increase of NREM sleep, further suggesting that alteration in cytokine concentrations may underlie sleep alterations observed in HIV infections (413).

In human African trypanosomiasis (HAT), a parasitic disease also known as sleeping sickness, circadian sleep-wake disruption is characteristic, with sleep and wake periods distributed throughout the 24-h day (77, 83). In comparison to Encephalitis Lethargica, there is a certain overlap of brain structures affected by both disorders, including hypothalamic centers, where many sleep-wake regulatory circuits are located (for comprehensive reviews on the pathophysiology of Encephalitis Lethargica and HAT, see Refs. 361, 570). It has been shown that the parasite trypanosoma can produce PGs, including PGD_2_ with known somnogenic properties, and triggers the release of pro-inflammatory cytokines (298). Increased PGD_2_ levels have been found in the CSF of patients in the late stages of the disease, further suggesting that the PGD_2_ system contributes to hypersomnia in HAT (435). In the rat model of HAT, it has been reported that sleep alterations, including circadian sleep-wake disruption, sleep fragmentation, and decreased REM sleep latency, start early after infection and become increasingly severe at the time of neuroinvasion of the parasite (170).

Sleep disturbances are also commonly observed in chronic infection with the hepatitis C virus (HCV), with more than 50% of patients reporting fatigue, daytime sleepiness, and poor sleep quality (reviewed in Ref. 388). Based on objective sleep assessment using actigraphy, increased nocturnal wake time and reduced sleep efficiency have been found in women infected with HCV (232). Of note, sleep alterations in this study were independent of the viremic state, suggesting that the sleep-immune relationship in HCV is complex. In patients with an infectious illness linked to the Epstein Barr virus (EBV), such as infectious mononucleosis, self-reported sleep duration was markedly increased (349) and debilitating daytime sleepiness persisted over many years (218). T-cell activation and cytokine production, in particular IFN-γ and TNF, are thought to be responsible for the clinical symptoms of infectious mononucleosis (21, 194, 581). However, whether such immune alterations also contribute to the observed sleep changes has yet to be addressed.

Long-lasting consequences of an infectious challenge may also play a pathogenic role in ME/CFS, where a viral etiology is still debated (29). Subjective complaints of disturbed, nonrestorative, and unrefreshing sleep are among the many symptoms in ME/CFS. Based on conventional sleep parameters assessed by PSG, several, though not all, studies reported increased sleep onset latency, increased number of nocturnal awakenings, and reduced sleep efficiency in ME/CFS patients compared with healthy control participants (for review, see Ref. 269). Using nonconventional EEG sleep analysis approaches, a few studies also found alterations in SWA, such as lower SWA in the ultra-low delta range (i.e., slow oscillatory activity) (330) or a blunted SWA response to a sleep onset delay in ME/CFS patients (17), as well as alterations of cyclic alternating patterns, indicating increased sleep instability (219). A number of immunological and inflammatory parameters have been found to be abnormal in ME/CSF, including altered cytokine profiles, increased numbers of activated cytotoxic CD8 T cells, reduced T-cell responses to mitogens, and impaired function of NK cells; however, findings across studies are often conflicting (reviewed in Ref. 342). With respect to circulating cytokine profiles, a recent systematic review reported an increase in transforming growth factor (TGF)-β as the most consistent finding across studies (59). With the use of a comprehensive immune profiling approach in ME/CSF patients, elevated TGF-β levels were confirmed, in addition to findings of lower resistin levels (390), a protein participating in the inflammatory response and able to activate TNF in monocytes and macrophages (for review, see Ref. 187). In the same study, 17 cytokines, most of them pro-inflammatory, correlated with symptom severity in ME/CFS (390), while another recent investigation reported an activation of a number of pro- and anti-inflammatory cytokines during the early stage of the disorder only, suggesting that immunopathology in ME/CFS fluctuates depending on disease duration (242). The complexity of the relationship between immune parameters and symptoms in ME/CSF is also suggested by a recent study showing that peripheral inhibition of IL-1 by the IL-1ra anakinra over a 4-wk period did not lead to clinically significant reduced fatigue in women with ME/CSF compared with a placebo group (473). Unfortunately, reports of sleep disturbance and nonrestorative sleep were not solicited in this study.

#### 2. Chronic inflammatory diseases

Sleep disturbances are also commonly found in diseases with inflammatory pathophysiology, such as inflammatory bowel diseases (IBD). IBD are chronic inflammatory disorders of the intestine with unknown pathogenesis, including Crohn’s disease and ulcerative colitis. In a recent study, half of the IBD patients reported sleep disturbances as assessed by the PSQI (550), with strongest associations in patients with active disease (10, 209). Based on data from the Nurses’ Heath Survey with over 400 IBD cases, self-reported sleep duration of <6 h and >9 h per night increased risk of ulcerative colitis (11). The association between IBD and sleep disturbances is thought to be bidirectional, such that increased disease activity leads to sleep disturbances, which in turn exacerbates inflammation. Supporting such an association, subjective sleep quality [assessed with the National Institutes of Health Patient-Reported Outcome Measurement Information System (PROMIS)], was inversely correlated with CRP levels in well-phenotyped IBD patients (584). In a prospective cohort, anti-inflammatory therapies with anti-integrin (vedolizumab) and anti-TNF agents (infliximab or adalimumab) resulted in improved sleep quality (assessed with PROMIS) within 6 wk of therapy initiation (527). This suggests a modulatory influence of cytokines on subjectively assessed sleep quality. In mice, using a dextran sodium sulfate colitis model, a worsening of colonic inflammation and colitis severity has been observed following chronic intermittent sleep deprivation (534), demonstrating a causal involvement of sleep in aggravating this disease.

One of the most extensively investigated chronic inflammatory disorders with respect to sleep is RA. This chronic inflammatory autoimmune disease primarily affects joints, leading to pain and potentially to joint destruction and deformation, if left untreated. About 50% of RA patients report sleep disturbances, including symptoms such as difficulties falling asleep and maintaining sleep, nonrestorative sleep, and excessive daytime sleepiness (reviewed in Ref. 456). Based on objective PSG and actigraphy assessment, various indicators of disrupted sleep have been reported in RA patients compared with healthy control participants, including increases in α-EEG intrusion, arousals, sleep stage shifts, sleep fragmentation, and nocturnal wake time (54, 160, 237, 351, 472). Alterations in sleep architecture, such as changes in the amount or distribution of SWS, have often not been found or show little consistencies between studies (reviewed in Ref. 53). A few studies investigated the link between sleep disturbances with immune alterations in this disorder. The immunopathology in RA is reasonably well understood and is characterized by inappropriate production of various cytokines, in particular TNF, among several other immunological abnormalities (94, 188). In a recent investigation, cellular inflammation, as indicated by spontaneous or stimulated production of TNF or IL-6 by monocytes, was higher in RA patients compared with matched healthy controls, and showed a complex time-of-day-dependent association with sleep efficiency and SWS amount. For example, higher sleep efficiency was associated with lower stimulated monocytic TNF production in the morning, but with higher production when assessed in the evening. As stated by the authors, these findings may suggest a feedback loop between sleep parameters and cellular production of pro-inflammatory cytokines (54). In a large cohort of over 8,000 patients from the National Data Bank of Rheumatic Diseases, self-reported sleep disturbances, including trouble falling asleep, trouble getting back to sleep, and not getting enough sleep to feel rested, did not differ between those who received anti-TNF therapy and those who did not (587). In contrast, treatment with anti-TNF therapy in RA patients with high disease activity resulted in an improvement of self-reported sleep quality, without affecting PSG-derived sleep parameters (283). However, at least three studies reported PSG-derived sleep improvements in response to anti-TNF therapy. In a noncontrolled study in RA patients with active disease, anti-TNF treatment (infliximab) resulted in decreased sleep latency, increased sleep efficiency, fewer sleep stage transitions, and increased REM sleep. These sleep improvements did not relate to joint pain amelioration, and therefore may independently reflect inhibition of raised circulating TNF levels (598). In another study in RA patients treated with anti-TNF therapy, PSG-assessed sleep improved, as indicated by increased sleep efficiency and less WASO (535). PSG-derived increases in sleep efficiency and total sleep time were paralleled by a reduction of CRP levels in patients with active disease treated with anti-TNF therapy (141). Furthermore, treatment with an IL-6 receptor inhibitor (tocilizumab) in RA patients with active disease improved self-reported sleep quality (assessed by the PSQI) and daytime sleepiness. Observed sleep improvements could not be explained by a reduction in disease activity, further suggesting that the cytokine-sleep relationship is not secondary to classical disease symptoms, in particular pain (196). To summarize, investigations on cytokine inhibition in RA suggest that TNF, in particular, plays a role in sleep modulation, although the independence of this association from other factors, such as disease activity, pain, and comorbid disorders has often not been addressed in detail. Subjective and objectively verified sleep disturbances have been also reported in a number of other rheumatic disorders, including Sjögren’s syndrome, systemic lupus erythematosus, systemic sclerosis, osteoarthritis, and other chronic diseases with an inflammatory involvement, such as migraines. Comprehensive reviews can be found in References 1, 53, 456.

Collectively, studies devoting attention to sleep disturbances in chronic infectious or inflammatory diseases demonstrate that sleep changes are common and can vary greatly, including shortened, prolonged, disrupted, or displaced sleep. Such variations in the sleep response likely depend on the type of immunopathology involved in the disease, disease activity, symptom severity, and disease stage. To date, only a few studies have specifically addressed the pathophysiological mechanisms responsible for sleep disturbances in chronic infectious or inflammatory diseases. This may relate to several challenges in this area of research: chronic infectious or inflammatory diseases frequently occur in conjunction with comorbid medical conditions, including depression, anxiety, obesity, cardiovascular disease, and pain, all of which alter sleep and immune system markers and thereby increase the complexity of the sleep-immune relationship. Furthermore, the immunopathology in chronic diseases may not be static over time. Depending on disease duration, the association between symptoms and immune parameters may differ in early versus late stages of a disease. Nonetheless, in light of basic research supporting a bidirectional relationship between sleep and the immune system (see next sections), it is most likely that such a link also exists in the context of chronic infectious and inflammatory diseases. However, the strength and independence of such an association from the many factors comorbid with chronic diseases (in particular pain) still needs to be determined.

## III. SLEEP EFFECTS ON HOST DEFENSE

### A. Experimental Studies Investigating Sleep Effects on Immune Parameters

A number of experimental studies have investigated the impact of experimental sleep manipulation on various immune parameters. These parameters include numbers of leukocytes and leukocyte subsets in the blood and various tissues, circulating cytokine levels, cytokine production in specific leukocytes, concentration of antibodies and complement factors in the blood, as well as functional aspects like cell cytotoxicity, to name only the most frequently investigated parameters. The main type of sleep manipulation is sleep deprivation, and the studies differ largely in the methods used to prevent sleep. Some studies induced total sleep deprivation; others partially deprived sleep, i.e., restricted sleep to a few hours per night or selectively suppressed REM sleep, using different methods that are more or less inherently stressful (TABLE 1). In addition, the duration of the sleep modification procedure differed largely between studies, ranging from only a few hours to several weeks. A limited number of studies employed an alternative approach to sleep deprivation. Some of those studies extended the opportunity to sleep [e.g., by a daytime nap (178–180), or by extending bedtime (i.e., increasing the nighttime sleep period) (103), see sect. V, *B* and *C*], while others intensified sleep [e.g., by acoustic stimulation (49)]. Because of the large methodological differences, it is difficult to compare across studies, and inconsistencies between studies are in part due to this methodological variety. TABLE 2 gives a broad overview of the research results and indicates how often a specific finding has been replicated. We define sleep manipulation of one day or less as acute sleep deprivation/restriction in contrast to protocols using more prolonged sleep manipulation. The idea behind this distinction is that experiments employing acute sleep manipulation are usually performed to assess the active role of sleep for the immune system (i.e., viewed from a sleep perspective) compared with a condition without sleep. In contrast, studies using prolonged sleep deprivation are intended to examine the impact of a lack of sleep on immune parameters. In TABLE 2, we therefore distinguish between acute effects of sleep (viewed from the sleep perspective) and effects of prolonged sleep loss (viewed from the perspective of sleep loss).

**Table 2.** Sleep effects on immune parameters

	**Acute Effect of Sleep**	**Effect of Prolonged Wakefulness**	**Reference Nos.**
***Leukocyte numbers in blood*** *(sect. III*B1*)*			
WBC	**↓↓↓** = = =	**↑↑↑** =	3, 67, 69, 103, 122, 152, 178, 224, 234, 254, 287, 326, 338, 469, 480, 580
Lymphocytes	**↓↓** = = = **↑**^(evening after normal sleep)^	**↑** = = =	3, 24, 67, 69, 103, 122, 152, 178, 224, 234, 251, 254, 326, 338, 480, 580
Monocytes	**↓↓↓** = = = **↑**^(4 days after partial SD)^	**↑↑↑** = =	3, 24, 67, 69, 103, 146, 147, 152, 178, 224, 254, 263, 287, 326, 480, 580
T cells, CD4 T cells, CD8 T cells	**↓↓↓** = = = **↑↑**^(mainly evening after sleep)^	= = =	3, 46, 67, 148, 152, 234, 251, 263, 418, 469, 480, 556, 580
B cells	**↓** = = =	**↑** = =	3, 67, 152, 234, 418, 480, 556
NK cells	**↓↓↓** = = **↑↑↑**	**↑** **↓**	3, 67, 152, 178, 234, 251, 263, 418, 556, 580
Neutrophils	**↓↓↓** = = = **↑**	**↑↑↑** =	67, 69, 103, 112, 122, 178, 287, 326, 338, 469, 480, 580
Basophils, eosinophils	= = =	= =	67, 152, 287, 338, 480, 580
***Cytokines/cytokine receptors*** *(sect. III*B2*)*			
*IL-6*			
Plasma/saliva levels	**↓↓** = = = **↑↑**	**↑↑↑** = = =	67, 104, 147, 180, 200, 223, 224, 236, 245, 248, 261, 334, 464, 480, 489, 506, 541, 547, 556, 562–564, 594
Production (intracellular or in supernatant)	**↓↓↓** = **↑**^(after partial SD)^	**↑↑** =	89, 147, 245, 256, 262, 264, 356, 512, 556
Tissue expression	**↓** =	**↑↑** = =	285, 290, 559, 604
*TNF*			
Plasma levels	**↓↓** = = = **↑**^(daytime after normal sleep)^	**↑↑↑** = = =	104, 146, 224, 236, 245, 248, 261, 334, 480, 489, 506, 541, 564, 594
Production (intracellular or in supernatant)	**↓↓↓** = **↑↑**^(mostly after partial SD)^	**↓** = = **↑**	24, 67, 146, 149, 256, 262, 264, 543, 552, 556
Tissue expression	**↓↓** = = **↑**	**↑↑** = =	18, 19, 162, 285, 290, 356, 553, 559, 604, 606
Soluble TNF receptor	= =	**↑** =	104, 222, 224, 506
*IL-1*			
Plasma levels	**↓↓** =	= = **↑↑**	171, 200, 236, 245, 386, 480, 541, 547, 594
Production (intracellular or in supernatant)	↓ = =	= = **↑↑**	24, 67, 103, 245, 552, 556
Tissue expression	**↓↓** = = **↑↑**	**↑↑** = =	18, 19, 162, 281, 290, 348, 356, 547, 553, 604
IL-1ra	**↓** =	**↑**	200, 224, 245
*IL-2*			
Plasma levels	=	= =	236, 248, 480, 547
Production (intracellular or in supernatant)	= = **↑↑**	**↓**	24, 62, 67, 149, 254, 552
Soluble IL-2 receptor	= =	**↓** **↑**	149, 222, 506
*IL-12*			
Intracellular production	**↓** **↑↑**		148, 323, 465
*IL-4, IL-10*			
Plasma levels	=	= = =	171, 236, 248, 480, 506, 547, 594
Production (intracellular or in supernatant)	**↓↓** =	=	24, 149, 323, 543
*Th1/Th2 ratio*			
Production (intracellular or in supernatant)	**↑ ↓**^(2nd half of night)^	**↓**	24, 149
***Immune cell activity and proliferation*** *(sect. III*B3*)*			
NK-cell activity	= **↑↑**	**↑** = **↓**	137, 152, 253, 254, 386
Lymphocyte proliferation	= = **↑↑**	**↑** = = **↓**	33, 62, 152, 243, 386, 482, 556, 580
Neutrophil function	**↓** = = **↑**	= =	112, 122, 421, 469
nTreg function	↑		62
***Antibodies, complement and other immune parameters*** *(sect. III*B4*)*			
Total IgG, IgM, or IgA antibody levels	**↓↓** = = **↑**	**↓** = = = **↑↑**	122, 171, 247, 418, 480, 596
Complement	**↓** = = **↑**	= = **↑**	247, 465, 480, 596
Adhesion molecules	**↓↓** = =		200, 489
Inflammatory gene expression		**↑↑**	6, 387

The number of icons represents the number of studies supporting the respective finding (3 icons, >3 studies; 2 icons, 2–3 studies; 1 icon, 1 study). Please note that the depiction of the studies is very simplified, as differences in designs are not considered. Most studies measured samples taken in the daytime after normal sleep or sleep deprivation, but not at night. Hence, some seemingly contradictory findings emerge because opposite results are found for nighttime and daytime values. The left column shows the acute effects of sleep (one night of sleep compared with one night of total/partial sleep deprivation), whereas the right column shows the effects of prolonged sleep loss (several nights with total or partial sleep deprivation). Refer to section III*A* for details. SD, sleep deprivation; WBC, white blood cells; NK cells, natural killer cells; IL-1ra, interleukin-1 receptor antagonist; nTreg, natural regulatory T cells.

There are also several studies that measured changes in immune parameters across 24 h without experimentally manipulating sleep. However, it is beyond the scope of the current review to elaborate on these studies. The interested reader is therefore referred to other recent reviews about circadian rhythms in the immune system (e.g., Refs. 95, 207, 494). Finally, several studies employed sleep deprivation in combination with exercise, psychological stress, or energy depletion (71, 220, 369, 402, 579, 588). Unless the effects of sleep were clearly distinguishable from those of the other manipulations (e.g., Refs. 122, 251, 469), results of those studies are not included in the following sections.

#### 1. Leukocyte and leukocyte subset numbers and distribution

In sleep studies involving healthy humans, the numbers of various leukocyte subsets in the blood are among the most often investigated parameters. Many leukocytes display strong migratory capacity and are constantly recirculating between various tissues and organs, using the blood and the lymphatic system as traveling routes. This migration leads to acute changes in the blood count of leukocyte subsets and has a very pronounced circadian component (95, 494). On top of this strong circadian rhythm, sleep exerts an overall weaker, but still significant, influence on leukocyte numbers in blood. Several studies in humans have found a reducing effect of sleep on total leukocyte count when compared with total sleep deprivation or sleep restriction for either only a few hours or up to several days (67, 69, 152, 178, 287, 326, 338, 480). Other studies failed to detect this reduction (3, 103, 122, 224, 234, 254, 469, 580). Such discrepancies could be due to the fact that most studies assessed cell numbers only at a single or just a few time points across the day, and often not during sleep. This reduces the chances of detecting transient changes in cell counts. Of note, none of the studies found that sleep increases leukocyte numbers. Therefore, it may be concluded that sleep reduces numbers of leukocytes in the blood, albeit only transiently, which enhances the risk of missing those changes.

Similarly, total monocyte counts, lymphocyte counts, and the major lymphocyte subsets (i.e., B cells, CD4 and CD8 T cells, NK cells), have been found to be either reduced by sleep (46, 67, 148, 251, 254, 480, 556, 580) or unchanged (3, 69, 103, 122, 152, 178, 224, 234, 263, 338, 418, 463, 469) when compared with acute or prolonged sleep reduction in humans. However, there are some reports of increased numbers of lymphocyte subsets (i.e., CD4 T cells, CD8 T cells, and NK cells), particularly when measured in the evening after a regular night of sleep (152, 234, 418, 556, 580). As suggested by Born et al. (67), these increases could reflect a homeostatic response, as numbers of lymphocytes and various subsets were acutely reduced during sleep, but increased in the afternoon or evening following regular sleep compared with nocturnal wakefulness. As for granulocyte subsets, basophils and eosinophils were not found to be affected by sleep (67, 152, 287, 338, 480, 580). In contrast, several studies found that sleep reduces neutrophil counts (69, 112, 178, 287, 326, 338, 480, 580), although again some studies did not find any changes (67, 103, 122, 469).

Since most studies have reported that sleep reduces numbers of various leukocyte subsets in the blood, the question concerning the fate of these cells arises. An impact on cell proliferation is unlikely, given that the effects of sleep already emerge within 3 h of sleep, or even earlier, whereas changes in proliferation would take significantly longer to become evident (67, 148). Therefore, a redistribution of the cells from the circulation to different tissues and organs is most probable. Animal studies have assessed cell numbers in different tissues, mainly primary and secondary lymphoid organs, after experimental manipulation of sleep. However, a unifying picture cannot easily be drawn. One study in mice found an increase in total splenic cells during sleep compared with prolonged wakefulness, but leukocyte numbers in the blood were unchanged, which contrasts with the findings in humans (346). Other rodent studies found an increase in numbers of leukocytes and lymphocytes in the blood after normal sleep compared with forced wakefulness, whereas there were no changes in cell numbers in the lymph nodes, spleen, or bone marrow (171, 217, 596, 597). Using a skin allograft model, Ruiz et al. (479) recently showed that numbers of total T cells, CD4 T cells, and CD8 T cells in the lymph nodes and spleen were higher after undisturbed sleep than after prolonged sleep restriction, which was accompanied by an increased allograft rejection. Interestingly, whereas Zager et al. (596) found no immediate changes in lymphocyte numbers in secondary lymphoid organs after acute sleep deprivation, they showed increased lymphocyte counts in lymph nodes after recovery sleep. This finding suggests that the typical increase in sleep intensity following sleep deprivation can lead to an accumulation of lymphocytes in this tissue. The most consistent findings with respect to the effect of sleep on leukocyte distribution have been observed for B cells and NK cells, which were shown to be increased in numbers in the murine spleen after normal sleep (137, 346, 360, 597). However, to summarize, findings on the effects of sleep on leukocyte distribution are far from conclusive. This may partly be due to methodological issues; for example, some studies investigated only one or a few tissues in parallel, and studies applying in vivo tracking of the cells are missing so far. In addition, comparing the findings obtained in animal models with those from human studies is difficult. Animal studies mostly investigated cell numbers in different tissues, but not in the blood, which is the compartment on which studies in healthy humans have so far focused on. Therefore, it has not been possible yet to relate changes in cell numbers in the blood circulation to changes in other tissues. Furthermore, redistribution kinetics of leukocytes may be different in humans and animals given the large differences in body size between species. Consequently, while sleep appears to affect the circulating number and migration of almost all leukocyte subsets, the question as to where leukocytes redistribute during sleep remains largely open.

#### 2. Cytokine levels, cytokine production, and cytokine receptors

The most often investigated cytokines in the context of sleep research are the acute phase cytokines IL-6, TNF, and IL-1. Fewer studies have investigated the role of sleep in modulating other cytokines, such as IL-2 and IL-12 (which are essential for the formation of adaptive immunity), the anti-inflammatory cytokine IL-10, or the Th2 cytokine IL-4. There are several ways to measure cytokines. Basal levels can be measured in blood (plasma) or saliva, although the concentration of most cytokines in healthy participants is usually very low and not always detectable. Cytokine production by leukocytes (or other cells/tissues) can also be measured, either unstimulated or after stimulation (e.g., with PAMPs like LPS), intracellularly on a single-cell level using flow cytometry or in bulk analyses across numerous cells at protein or mRNA level, or in the supernatant of in vitro cultures. It seems that sleep has a differential effect on cytokines depending on which type of cytokine is assessed through which method and at which time of the day. Because of the large methodological differences between studies, findings on the effects of sleep on cytokines are very mixed, especially for the effects of sleep when compared with acute sleep loss.

##### A) IL-6.

Sleep can show enhancing (180, 200, 464), decreasing (541, 562, 563) or no effects (67, 104, 147, 224, 261, 489) on IL-6 levels in plasma or saliva when compared with one night with no or restricted sleep in healthy participants. Of note, measurements of IL-6 in saliva do not seem to reflect systemic levels of this cytokine in plasma (127), but differences in the body fluid used to measure IL-6 are not sufficient to explain the discrepancies between these studies. More consistent findings are observed if sleep is diminished for longer than one night: plasma levels of IL-6 or CRP (which is produced by the liver in response to IL-6 secretion) mostly increase in such conditions, likely reflecting a systemic, unspecific inflammatory response to prolonged sleep loss (223, 506, 556, 564) (see also sect. IV). These increases are in line with results of enhanced IL-6 levels in animal studies, all of which used prolonged sleep deprivation protocols (245, 541, 594). On the other hand, there are also studies reporting no effect of prolonged sleep restriction on IL-6 or CRP levels in humans (69, 334, 480, 506) and animals (236, 248), suggesting that the effect is transient and may not be detectable if samples are only measured at a single time point.

Intracellular IL-6 production was not altered acutely during sleep compared with nocturnal wakefulness (147), but was increased in the morning after one night of partial sleep loss in healthy humans (89, 256, 262, 264). This effect was also found at the mRNA level, measured in peripheral blood lymphocytes of mice after 36 h of sleep deprivation (245). Increased stimulated intracellular IL-6 production was also shown after prolonged sleep restriction for several days in humans (512, 556), although one of the studies found the increase only at the mRNA, but not the protein level (556). Animal studies measuring IL-6 protein or mRNA expression in different tissues, especially the brain, found either increases (285, 290, 356, 559) or no changes (285, 553, 604) after shortened sleep.

##### B) TNF.

Plasma levels of TNF were mostly unchanged during or on the day after acute sleep deprivation/restriction in healthy humans (224, 261, 334, 480, 489, 506). One study found TNF levels to be reduced during sleep, but to be enhanced on the day after normal sleep compared with a nocturnal vigil (146), potentially reflecting a homeostatic regulation of this cytokine. However, another study found reduced levels in the evening following regular sleep (104). As to prolonged sleep curtailment, a mild reduction of sleep to 6 h per night for 1 wk led to an increase in TNF plasma levels in one study (564), although other studies in humans found no effect after prolonged sleep deprivation or restriction (334, 480, 506). Similarly, some rodent studies found enhanced plasma levels of TNF following 36 and 72 h of sleep restriction (245, 594), whereas other studies reported unchanged TNF plasma levels after sleep restriction for 48 h and 8 days in mice and rats (236, 541). One recent study found increased TNF levels after 10 days of sleep restriction in C57BL/6 mice, but not in BALB/c mice (248). This finding corresponds to the results of the two studies mentioned above, which used these different strains (236, 245), and might at least partly explain the divergent findings. Overall, compared with the results for IL-6 levels in plasma, which mostly showed an increase with prolonged exposure to sleep deprivation or restriction, possible increments in TNF levels after longer sleep deprivation seem to be more difficult to detect in humans and animals.

Stimulated production of TNF in humans has been found to be acutely reduced during sleep compared with nocturnal wakefulness when measured in the supernatant (552) or intracellularly in CD8 T cells (149), but increased when measured in monocytes (146). Other studies, however, failed to find an acute effect of sleep on TNF production assessed in the supernatant (67, 543). Following sleep deprivation in the first night half, TNF production in monocytes and TNF mRNA were shown to be increased in the morning (256, 262, 264). These findings stand in contrast to the sleep-induced increase in TNF production in monocytes reported by Dimitrov et al. (146). Hence, the findings of increased TNF production in the morning after sleep deprivation by Irwin et al. (256) may reflect a rebound effect of sleep, as participants were allowed to sleep in the second night half before blood was collected in the morning. Interestingly, Irwin et al. (256) found striking sex differences, with further increases of TNF in the evening in women, but decreases in men. After prolonged sleep restriction of more than one night, stimulated TNF production was reduced in the supernatant in one study in humans (556), but increased in another (24). Most animal studies measured TNF protein or mRNA levels in different tissues with or without stimulation using several methods for sleep manipulation, which renders the results difficult to compare with each other. Overall, most studies found either increased (18, 161, 285, 356, 553, 559, 606) or unchanged (19, 162, 290, 604) TNF levels in the various tissues, including the spleen, liver, brain, adipose tissue, and peritoneum after sleep loss. These findings are an important addition to the studies in humans, in which cytokine levels were mainly assessed in the blood circulation.

Finally, a handful of studies in healthy humans investigated the effect of experimental sleep manipulation on the soluble TNF receptors I and II, which act as inhibitors and regulators of TNF signaling. One night without sleep did not change the plasma levels of these receptors (104, 222). In contrast, prolonged total sleep deprivation of four consecutive nights increased levels of the soluble TNF receptor I (506), which may reflect a homeostatic response to increased TNF production by leukocytes; this, however, was not investigated in the study.

##### C) IL-1.

As for plasma levels of IL-1, two human studies found an acute increase in IL-1 following one night of sleep deprivation (200, 386), while another study employing either two nights of total sleep deprivation or four nights of REM sleep deprivation did not find an effect of sleep in healthy participants (480). In rodents, three studies found increases in IL-1 plasma levels after sleep deprivation (171, 245, 594), although other studies failed to substantiate these findings (236, 541, 547), which is similar to the mixed findings in humans. IL-1 production and mRNA expression were mostly found to be unchanged by both acute and prolonged sleep manipulation in healthy humans (24, 67, 103, 556). However, an increase at the mRNA level after prolonged sleep restriction has been reported in humans (556), as well as in animal studies measuring gene expression in peripheral blood lymphocytes (245), and in the spleen, liver, CNS, heart, and adipose tissue (18, 162, 281, 290, 348). Some animal studies found unchanged (19, 162, 356, 553, 604) or reduced (19, 547) IL-1 levels in different tissues other than the blood after sleep loss. Although findings are mixed, none of the human and animal studies measuring IL-1 levels either in plasma or intracellularly in circulating leukocytes reports a reduction of this cytokine following acute or prolonged sleep deprivation or restriction. The receptor antagonist IL-1ra, which is a natural inhibitor of IL-1 signaling, was increased after sleep deprivation in humans and animals (200, 245). Similarly to the soluble TNF receptor I, the increase in IL-1ra might reflect a homeostatic response following increased IL-1 levels.

##### D) OTHER CYTOKINES.

Other cytokines have been reported less frequently, although they are no less physiologically significant. IL-12 and IL-2, for example, are essential for the formation and maintenance of adaptive immunity, such as in the context of a vaccination response, which will be discussed in section III*B*. Studies attempting to measure IL-2 levels in plasma have failed to find an effect of sleep manipulation in humans (480) and animals (236, 248, 547). In contrast, stimulated IL-2 production seems to be acutely enhanced by sleep in healthy humans (67, 254, 552), although other studies did not find such an effect when assessing stimulated IL-2 production specifically in CD4 T cells (62, 149). Five nights of partial sleep deprivation led to a reduction of stimulated IL-2 production in healthy humans (24), which is in line with the studies manipulating sleep for only one night. The effect of sleep on the soluble IL-2 receptor, which may be used as a surrogate marker of T-cell activation (392), was also investigated. Two studies in humans found no change in this receptor after one night without sleep (149, 222), whereas another study revealed an increase across several days of sleep deprivation (506). In contrast, one recent animal experiment showed a reduction following prolonged sleep deprivation in a skin allograft model (479). As for IL-12, two human studies found a striking increase of IL-12 producing cells during sleep compared with nocturnal wakefulness (148, 323), although this increase seems to be reversed when the cells are primed with IFN-γ before LPS stimulation (465).

A few studies have also investigated the impact of sleep on the anti-inflammatory cytokine IL-10 and the Th2 cytokine IL-4. Studies in both humans and animals did not find changes in plasma levels of IL-4 and IL-10 after sleep manipulation, although these cytokines are often below detection limits without stimulation (171, 236, 248, 480, 506, 547, 594). In contrast, human studies measuring stimulated cytokine production found a sleep-dependent drop in IL-10 production by monocytes (323) and in IL-4 production by CD4 T cells (149) if assessed at a cell-subset specific level in whole blood, but not if measured in a cell-subset specific level in isolated T cells (61), or in the supernatant of whole blood or peripheral blood mononuclear cell (PBMC) cultures (24, 543). The study by Dimitrov et al. (149) found an acute rise in the Th1/Th2 cytokine ratio (measured as the ratio of IFN-γ to IL-4) during early sleep compared with nocturnal wakefulness. This change, however, reversed in the second half of sleep. After 5 days of sleep restriction, Axelsson et al. (24) observed a shift in the Th1/Th2 cytokine balance (measured in this study as the ratio of IL-2 to IL-4) towards Th2 activity that was strongest during daytime after sleep restriction in humans. These findings suggest that sleep, and especially the early sleep period, is responsible for establishing a predominant Th1 cytokine response.

Altogether, the results on the impact of sleep on cytokine levels and production are complex, which is not only due to the differences in the methods used for inducing sleep deprivation mentioned above, but also because of methodological differences in the measurement of cytokine production. A major problem with the assays usually employed for measuring cytokine production is that they do not reflect normal physiology. Cells are often incubated for several hours and stimulated with high, non-physiological doses of stimulants. Given that the effects of sleep on cytokine production are potentially mediated by humoral factors (322), using isolated cells instead of measuring cells in whole blood (i.e., their natural physiological environment) hardly reflects the real in vivo situation. Another important issue is that results can be biased, as sleep does not only affect cytokine production itself, but also the numbers of circulating leukocytes that produce these cytokines. A selective extravasation of certain leukocyte subsets can therefore have an impact on the results of cytokine measurements. Furthermore, there are also other sources of cytokine production besides immune cells, such as endothelial cells (see TABLE 3 for an overview of sources of production of humoral immune molecules). Hence, it is important to view the effects of sleep on cytokines in the context of the potential sources producing the cytokine of interest. Cytokines are also often produced only locally and for a short period, and have short half-lives, which makes it difficult to detect changes, especially when measured in the blood. Finally, many studies did not collect blood during the sleep manipulation itself, but rather on the day after, and only at single or very few time points. Being a homeostatic system, the healthy body is expected to attempt to reach a homeostatic state, thus counteracting any effects occurring acutely during sleep deprivation. It is therefore not surprising that the observed effects of sleep can differ depending on the exact time point of measurement. Nonetheless, in general, it seems that sleep acutely favors pro-inflammatory and Th1 cytokine production over anti-inflammatory and Th2 cytokine production, with both being tightly regulated by a homeostatic response that reverses this effect at later time points (e.g., during the second half of sleep or the daytime period in humans). This delicate balance can be disturbed following prolonged reductions in sleep duration, which may lead to a chronic imbalance favoring a pro-inflammatory response that is likely to have deleterious health consequences over the long term (see sect. IV).

**Table 3.** Humoral immune molecules and their cellular sources of production

**Molecule**	**Cell Source**
IL-6	Monocytes, macrophages, T cells, B cells, granulocytes, mast cells, thymocytes, endothelial cells, fibroblasts, smooth muscle cells, chondrocytes, osteoblasts, glia cells, keratinocytes, pancreatic cells
TNF	Monocytes, macrophages, CD4^+^ T cells, B cells, neutrophils, NK cells, mast cells, fibroblasts, astrocytes, microglia, endothelial cells, smooth muscle cells, intrinsic renal cells, keratinocytes
IL-1	Monocytes, macrophages, lymphocytes, neutrophils, keratinocytes, microglia, megakaryocytes, fibroblasts, synovial lining cells, DCs, endothelial cells, smooth muscle cells
IL-1ra	Monocytes, macrophages, neutrophils, fibroblasts, endothelial cells, epithelial cells, keratinocytes, hepatocytes, astrocytes, bone marrow cells
IL-2	CD4^+^ and CD8^+^ T cells, DCs, NK cells, NKT cells, mast cells, innate lymphoid cells
IL-12	Monocytes, macrophages, neutrophils, microglia, DCs, B cells, NK cells, keratinocytes
IFN-γ	NK and NKT cells, macrophages, Th1 cells, cytotoxic CD8 T cells, B cells, DCs, neutrophils, endothelial cells
IL-4	Th2 cells, basophils, eosinophils, mast cells, NKT cells, γ/δ T cells, monocytes/macrophages, neutrophils, B cells
IL-10	T cells, B cells, monocytes, macrophages, DCs, mast cells, thymocytes, keratinocytes
Immunoglobulins	B cells
Complement proteins	Hepatocytes, endothelial cells, epithelial cells, neutrophils, mast cells, monocytes, macrophages, DCs, lymphocytes

NK cells, natural killer cells; DCs, dendritic cells; NKT cells, natural killer T cells; Th1, type 1 helper T cells; Th2, type 2 helper T cells. See References 7, 344 for further details on cellular sources of cytokine and complement production, respectively. Note that this table includes only the most commonly reported cellular sources of production.

#### 3. Immune cell activity and proliferation

Besides measuring leukocyte numbers and cytokine production, the impact of sleep on functional parameters, especially on NK-cell activity and lymphocyte proliferation, has also been investigated. During both the night of and the morning after total sleep deprivation or restriction, NK-cell activity was reduced in healthy humans (253, 254, 386), suggesting a supportive effect of sleep on this functional immune parameter. In contrast, no change was evident in the evening following sleep deprivation for one night (152). Interestingly, this latter study found increased NK-cell activity in the evening after two nights without sleep, possibly reflecting a slowly evolving counterbalancing response to NK-cell activity reductions reported during and in the morning after acute sleep loss (253, 254, 386). However, differences in the findings between these studies could also be due to differences in the time the assays were performed (morning vs. evening). One study in mice found a reduction in the cytotoxicity of NK cells after 24 and 48 h of sleep deprivation, which was not evident after a more prolonged deprivation of 72 h (137). It thus seems that sleep is important for optimal NK cell functioning, since acute deprivation or restriction of sleep may transiently dampen this function in humans and mice. However, if sleep deprivation or restriction is prolonged, adaptive processes of NK-cell activity may commence, as suggested by an increase or normalization, rather than further reduction, of the response.

Similarly complex is the impact of sleep on lymphocyte proliferation. Again, during and in the morning or afternoon following acute sleep deprivation of a single night in healthy humans, mitogen-stimulated cell proliferation was reduced (62, 386, 580), whereas no changes were detected in the evening after one and two nights of total sleep deprivation (152). However, following several nights with restricted sleep, an increase in PBMC proliferation was reported (556). Hence, as for NK-cell activity, adaptive processes (over-)compensating for the effect of sleep loss seem to occur. Animal studies have reported reductions in lymphocyte proliferation after sleep deprivation for 48 h and 72 h, but not 24 h (243). However, other experiments failed to find such an effect after prolonged sleep deprivation of 72 h or several days (33, 482). Overall, human and animal experiments suggest a general supportive influence of normal sleep on lymphocyte proliferation, although the effects may depend on the duration and type of sleep loss. Further research is needed to better understand adaptive or compensatory processes that may take place with more chronic forms of sleep loss.

A few human studies have investigated neutrophil functions, mostly by assessing markers of degranulation. Whereas two studies did not find an effect of sleep on neutrophil function (421, 469), one study reported a transient increase in neutrophil degranulation after one, but not two nights without sleep (122). In contrast, ROS production in neutrophils was reduced after one night without sleep, but no change was evident in phagocytosis or the percentage of phagocytizing neutrophils (112). Finally, the suppressive capacity of natural Tregs was shown to be higher during sleep as compared with a nocturnal vigil (62). However, because of the very low number of studies, the results concerning neutrophil and Treg cell function have to be interpreted with caution.

#### 4. Circulating levels of antibody classes, complement factors, and other immune parameters

Research on the effect of sleep on the humoral immune system (including total IgG, IgM, and IgA antibody levels and complement factors) is very scarce so far, and results should therefore be considered as preliminary. One study found increases in circulating IgG, IgM, and IgA levels in the morning after one night of total sleep deprivation in humans (247), which might reflect a change in the binding or consumption of antibodies rather than a de novo synthesis. Two other studies could, however, not replicate this finding (418, 480). Following four days of selective REM sleep deprivation, circulating IgA, but not IgG and IgM, levels were reduced (480). Another study found an increase in secretory IgA in saliva following one and two days without sleep; however, this might reflect an accumulation of the antibody due to a decreased saliva flow rate (122). Animal studies have found enhanced levels of circulating antibodies, especially for IgM, after sleep restriction for 10 days or longer, but not after shorter periods (171, 596). Overall, results so far are divergent, and the functional significance of changes in antibody levels is difficult to assess.

The complement system consists of several plasma proteins that are produced in a cascade of sequential cleavages by proteases and supports several other immune parameters in attacking pathogens. Inappropriate activation of the complement system has been shown to play a deleterious role in a number of rare diseases (e.g., Neuromyelitis Optica), but also in more common diseases, including autoimmune (e.g., RA), neurodegenerative (e.g., Alzheimer’s disease), acute inflammatory diseases (e.g., sepsis), and various roles for the complement system have been proposed in cancer and cancer therapies (reviewed in Ref. 239). Presumably due to its large complexity, only a few studies have investigated the effect of sleep on this aspect of the immune system. The complement factors C3 and C5 were increased after acute sleep deprivation in one human study (247), whereas others found no change in C3, C4, or the receptors C3aR and C5aR after either acute total sleep deprivation or prolonged REM sleep deprivation (465, 480). Complement activation, as indicated by plasma levels of C3a, was even found to be higher during sleep than during nocturnal wakefulness (465). The only animal study to our knowledge measuring effects of sleep on the complement system revealed a transient increase in C3 levels after 96 h of REM sleep deprivation, but not after shorter or longer periods of sleep restriction (596). C4 levels remained unchanged regardless of the duration of sleep manipulation.

Cell adhesion molecules are essential for cell-to-cell contacts, for example, between circulating immune cells and endothelial cells, which is an essential step in the migration process of leukocytes. These molecules can be found as soluble forms in the blood or expressed on the surface of cells. Two human studies investigated the impact of sleep on circulating levels of three different soluble adhesion molecules: intercellular adhesion molecule (ICAM)-1, vascular adhesion molecule (VCAM)-1, and E-selectin (200, 489). E-selectin and ICAM-1 levels were increased following one night without sleep, whereas VCAM-1 levels remained unchanged. The increases were interpreted as indicators of enhanced pro-inflammatory processes and endothelial activation, which could be linked to the development of cardiovascular disease (489) (see also sect. IV). Another study measured the expression of L-selectin and the integrin macrophage-associated antigen (Mac-1) on monocytes and lymphocytes (463). It found that the increase in the percentage of Mac-1-positive lymphocytes observed during a night with undisturbed sleep was prevented by depriving participants of sleep between 11 PM and 3 AM. This was interpreted as a reduced ability of the cells to migrate to sites of infection after sleep restriction. In contrast, sleep restriction enhanced the nocturnal increase of L-selectin-positive lymphocytes and monocytes that was evident during normal sleep. The lower number of L-selectin-positive cells during sleep compared with partial sleep deprivation was interpreted as an enhanced activation of immune cells during sleep, because L-selectin is shed following cell activation. However, a reduced expression could also be interpreted as a lower migration potential of the cells, as L-selectin is necessary for the first step in the migration cascade, which includes the slow rolling of the cells along the vessel walls. The results are complicated further by the fact that those cell subsets with a higher expression of L-selectin and Mac-1 might leave the bloodstream faster than cell subsets with lower expressions. Therefore, the reduced number of L-selectin-positive leukocytes during sleep could simply reflect a selective extravasation of cell subsets with a high expression of this molecule. Although the authors’ interpretations of the results are in line with the notion that sleep enhances the migration capacity of leukocytes and their activation, the study is also important in showing that findings on the cellular expression of molecules should be interpreted with caution.

Finally, another more general approach to investigate the impact of sleep on the immune system is to measure the change in global gene expression in leukocytes after sleep manipulation. The findings of human studies using this technique are generally in line with other results showing that sleep loss for longer periods enhances inflammatory pathways (6, 387) and can reduce the functional capacity of leukocytes (433). In addition, sleep restriction has been found to change the circadian rhythm of genes associated with immune and inflammatory responses; these perturbances in rhythmicity are likely to be also associated with health problems related to sleep disturbances (15, 387) as described in section IV.

### B. Sleep Effects on Immunological Memory

#### 1. Vaccination studies

The findings summarized in the previous section show that sleep affects a variety of immune parameters in the steady state. However, an effective immune response relies on the complex interplay between several immune cells and mediators. Therefore, it is essential to also consider the impact of sleep on an ongoing immune response. Vaccination is an optimal experimental model to tackle this question, as it mimics an infection and can be delivered at defined points in time to healthy humans. In contrast to the plethora of data on the impact of sleep on isolated immune parameters, only a few studies have investigated the direct effect of sleep on the immune response to vaccination. The very first human study in this context examined the influence of restricting bed time to 4 h per night for 4 days before and 2 days after vaccination against influenza. Influenza-virus-specific antibody titers measured 10 days after vaccination were more than doubled in participants that were allowed to keep their usual bed time of 7.5–8 h compared with those with restricted sleep (522). Further studies showed that also a single night without sleep on the night following vaccination against hepatitis A, hepatitis B, and H1N1 (swine flu) reduces the antigen-specific antibody response (which reflects immunological memory) to a similar extent (34, 320, 325). The only study that measured the effect of sleep on cellular immune parameters showed that sleep also enhances the number of antigen-specific CD4 T cells, as well as their production of the Th1 effector cytokine IFN-γ (320). These results demonstrate that sleep not only supports the memory phase of the immune response to vaccination, but also the effector phase. Of note, three participants from the Wake group had to receive an additional vaccination because their titers were below levels for clinical protection, whereas all participants from the Sleep group had sufficient protection, showing the clinical significance of the findings. All vaccines that were employed in these human sleep studies are known to induce mainly Th1-dependent IgG1 and IgG3 responses, which were selectively enhanced by sleep compared with nocturnal wakefulness (320). It is presently unknown, however, whether sleep likewise supports Th2 or CD8 T cell responses.

Of note, in all of these studies the magnitude of the sleep effect was similarly strong, i.e., on average sleep doubled the response to vaccination when compared with the wake or sleep-restricted groups. In contrast, the persistence of the sleep effect varied between experiments. Some studies failed to reveal any statistically significant differences between groups 4 weeks after vaccination (34, 522), whereas one study found a pronounced sleep effect even 1 year after the initial vaccination (320). In addition, those studies including male and female participants either found no sex differences (325) or a dramatic difference between sex, such that only men were affected by the sleep manipulation (34). These discrepancies might be related to differences in study designs and virus type, but on the whole, the few available studies in humans provide convincing evidence for sleep strongly supporting vaccination outcomes.

These experimental studies are also well in line with one prospective study that found habitual sleep duration (as measured by wrist actigraphy) to be related to the vaccination response against hepatitis B (447). Longer sleep duration in the 7 days surrounding the first of three vaccinations was associated with higher secondary antibody levels. When sleep duration was divided into the three categories ‟less than 6 h,ˮ ‟6–7 h,ˮ and ‟more than 7 hˮ of sleep per night, each additional hour of sleep was associated with an ~50% increase in antibody levels. Clinical protection status, which was assessed 6 months after the final vaccination, was achieved less frequently in participants with a short sleep duration, demonstrating the clinical significance of these findings. Sleep efficiency, as assessed by actigraphy, as well as self-reported sleep quality and duration, as assessed by sleep diaries, were not associated with the immune response. Interestingly, this study found no association between sleep duration around the time of the first vaccination and the primary antibody response, but only with the response after the second and the third vaccination. This stands in contrast to the experimental studies in which the effect of sleep was already evident for the primary immune response. However, habitual sleep duration measures the cumulative effect of sleep loss instead of the acute effect of a single night without sleep. As outlined below, SWS seems to be the most relevant sleep stage in mediating the acute effect of sleep (compared with one night without sleep) on the immune system. However, habitual short sleep is not necessarily associated with a reduction in SWS (574); therefore, other mechanisms might underlie the immune consequences of sustained reductions in sleep duration observed by Prather and colleagues (447). In section IV, we will take a closer look at the consequences of habitual short sleep, which include the development of low-grade inflammation. This chronic inflammatory state is associated with an increased risk for several diseases and with a decreased ability to mount adaptive immune responses (198), which might at least partly explain the above-mentioned findings of Prather and colleagues (317, 447).

Results from animal studies are overall less consistent. Most of the experiments are not readily comparable to the studies in humans, as they focused on the secondary immune response (i.e., sleep deprivation took place after a secondary challenge in previously immunized animals). In a very early study, mice that had been recently immunized with the influenza virus received a challenge, after which they were deprived of sleep by gentle handling for 7 h. In contrast to mice that were allowed to sleep, these animals had a lower influenza-specific antibody titer and could not clear the virus, thus resembling nonimmunized mice (73). A further study by the same group found comparable effects after immunization with sheep red blood cells (75). Other studies, however, did not find such dramatic effects of sleep or even found increased antigen-specific antibody titers after repeated sleep deprivation (466–468, 545). These discrepancies between studies could stem from differences in mice and virus strains, as well as differences in study design, especially in the timing of the sleep deprivation procedure in relation to the antigen challenge and the route of antigen administration. Renegar et al. (468) argued that in the early study by Brown and colleagues (73), animals had not developed complete immunity against the virus because of the different routes of antigen administration during immunization (orally) and later challenge (intranasally). Sleep deprivation might have impaired the migration of lymphocytes to the respiratory tract in that study, thus interfering with the development of mucosal immunity. In contrast, in other studies that did not show an impairing effect of sleep deprivation, robust immunological memory had already developed by the time of sleep manipulation (468). It therefore seems that sleep does not primarily affect existing immunity but rather supports the development of immunological memory to a new antigen. This conclusion is indeed well in line with the findings from the human studies in which sleep was manipulated at a time at which immunological memory was still developing.

Some of the studies in animals measured the effect of sleep deprivation on virus titers in previously nonimmunized control mice. However, this was measured only a few days after the challenge (i.e., during the expansion phase), but not during the memory phase, which did not allow for measurement of the antigen-specific antibody or T-cell response as it was done in the human studies. Most of these animal studies did not find an effect of sleep on virus titers (73, 467, 545), although one experiment reported reduced virus shedding in the respiratory tract after sleep deprivation (466). Hence, sleep does not contribute to the acute suppression of viral replication. As already suggested by Renegar et al. (468), sleep might rather support the migration of lymphocytes, thus supporting the formation of immunological memory. Indeed, the studies described in section III*A* showing a sleep-induced reduction in circulating lymphocyte numbers, together with other studies suggesting an accumulation of APCs and lymphocytes in lymphatic tissues during the rest phase, support this notion (63, 67, 143, 148, 169). Further mechanisms presumably mediating the positive effect of sleep on the development of immunological memory include an increase in the production of proinflammatory and Th1 cytokines, which are relevant for the early phase of immunological memory formation. Specifically, stimulated production of IL-12 and IL-2, which support the interaction between APCs and T cells and the subsequent Th1 cell differentiation and proliferation, respectively, was shown to be increased by sleep (67, 148, 323). The following section discusses possible mechanisms of the supportive effect of sleep on the vaccination response viewed from the perspective of a general role of sleep in supporting memory formation in the CNS as well as in the immune system.

#### 2. The memory function of sleep

**FIGURE 5.**
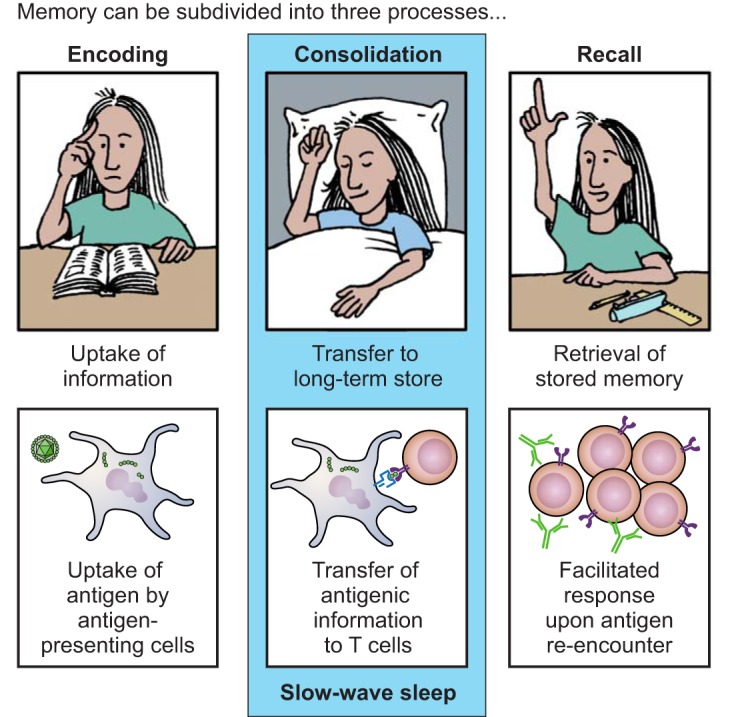
Sleep supports memory consolidation in the brain and the immune system. Memory processes in the brain are usually subdivided into three phases, which may also be used to categorize memory processes in the immune system. During the *Encoding* phase, the information to be remembered is taken up. In the immune system, this phase refers to the uptake of the pathogen by antigen-presenting cells (APCs). In the *Consolidation* phase, the initially labile information is transferred from the initial store to a long-term store. For memories in the brain, the information is moved from certain brain regions to others; for memories in the immune system, the information (i.e., the antigen) is transferred from APCs to T cells. During recall, the remembered information can be retrieved, which is represented in the immune system by the activation of memory T and B cells. For both the brain and the immune system, sleep and especially slow-wave sleep seem to be most important for the consolidation phase of memory processes. (Adapted from Tanja Lange.)

Although it is very likely that sleep does not serve only a single function, one assumed general role of this behavioral state is to support memory formation. For the CNS, this has been established in numerous studies (see Ref. 457 for a comprehensive review on that topic). Interestingly, sleep seems to play the same role for immunological memory, as shown by the vaccination studies described above. Comparing the role of sleep for memory formation in the CNS and the immune system might help to understand mechanisms underlying the supportive role of sleep in immunological memory formation. Although there are clear differences in memory formation in the CNS and the immune system, e.g., as to the forms of information storage, there seem to be some striking similarities. Analogously to the formation of memory in the CNS, memory in the immune system may be subdivided into three phases (FIGURE 5). *1*) Encoding: during the encoding phase, the information to be remembered first has to be sensed by our body. In the immune system, the relevant information is the antigen, which is sensed by APCs of the innate immune system. These cells take up the pathogen at the site of entry and represent an initial store for the antigenic information. *2*) Consolidation: during the consolidation phase, the sensed information is transferred from the initial store into a longer-lasting store. In the CNS, the initial and long-term stores are represented by different brain regions. In the immune system, the long-term store is represented by memory T and B cells and plasma cells of the adaptive immune system. The transfer of the information from APCs to T cells takes place in secondary lymphatic tissues, where APCs present antigens to T cells. Activated T cells then proliferate and differentiate into effector and memory cells and support the activation of B cells. *3*) Recall: this phase represents the retrieval of the memory for the encoded information. Memory recall in the immune system occurs mainly by activation of memory T and B cells upon re-encounter of the antigen and by antibody-driven effector mechanisms.

As stated in section III*B1*, sleep supports the formation of new immunological memory rather than affecting existing immunity. Thus, rather than supporting the recall phase, sleep seems to foster either the encoding or consolidation phase. Almost all of the human studies that demonstrated a supporting role of sleep in vaccination responses manipulated sleep in the night immediately after vaccination (34, 320, 325). It is therefore unlikely that sleep affected antigen uptake by APCs (i.e., the encoding phase), which should already have happened before the time of sleep. The sleep manipulation occurred within 24 h post-vaccination, i.e., the time window during which APCs and T cells interact in secondary lymphatic tissues. Therefore, sleep seems to support the consolidation phase of immunological memory formation. Several findings discussed in section III back up this idea. Sleep reduces the number of APCs and T cells in the blood, probably by redistributing these cells to lymph nodes (67, 143, 148, 169), thus increasing the chances of encounter of both cell types. In addition, sleep, by favoring pro-inflammatory and Th1 cytokine production, further supports the interaction between APCs and T cells and the subsequent differentiation and proliferation of Th1 cells, eventually leading to the formation of IgG1 and IgG3 antibodies (24, 67, 148, 254, 320).

In the CNS, sleep also supports memory formation during the consolidation phase. A further parallel between the effect of sleep on CNS and immunological memory is the predominant role of SWS. Although REM sleep might also support adaptive immune functions, the pro-inflammatory endocrine milieu present during SWS (including high levels of GH, prolactin, and aldosterone and nadir levels of cortisol) is optimally suited for promoting APC-T cell interactions. GH, prolactin, and aldosterone are known to support T-cell migration, the release of pro-inflammatory and Th1 cytokines, cell proliferation, and lymphocyte responses to a microbial challenge (43, 45, 48, 117, 126, 286, 355, 362–365, 380, 389, 478, 516, 518, 524, 575, 583, 601). On the other hand, low levels of cortisol during SWS could allow efficient APC-T cell interactions (157, 211, 417, 427, 438). In line with this idea, the amount of SWS and SWA in the slow oscillation range (i.e., slow frequencies of ≤1 Hz), as well as the accompanying increases in GH and prolactin and reduction in cortisol levels in the night following vaccination, correlated with the percentage of antigen-specific CD4 T cells measured up to 1 year later (320). Thus the general idea is that SWS, by inducing this unique endocrine constellation, supports the interaction between APCs and T cells, which results in a stronger immunological memory (578). Evidence for the association between SWS and the pro-inflammatory endocrine milieu was so far correlational in nature (98, 216, 521). However, a recent study showed that specifically enhancing slow oscillation activity during sleep in healthy participants intensified the hormonal milieu during SWS (especially by further increasing aldosterone and decreasing cortisol levels) and reduced circulating lymphocyte counts (49). These findings indicate that SWS actively induces these endocrine and immunological changes rather than being simply a correlate.

At present, little is known about sleep’s effect on the encoding phase of immunological memory. Two studies from the 1970s suggest that sleep improves processes of antigen uptake and phagocytosis (93, 420). However, since then, no studies have further studied the specific impact of sleep on the encoding phase of immunological memory formation. Measuring habitual sleep in the 7 nights surrounding a vaccination did not reveal any single night as uniquely predictive of the humoral antigen-specific response (447). However, this null effect might be due to a small variance between the individual nights within participants. Therefore, experimental studies that manipulate sleep in the night before vaccination are needed to unravel whether the encoding phase of immunological memory does also profit from adequate sleep.

To summarize, one important function of sleep and especially SWS might be to support the formation of memory in the two super systems, i.e., the nervous and the immune system, which allows an optimal adaptation of the organism to the environment. For the immune system, this has been demonstrated by several vaccination studies in humans, showing that sleep after vaccination doubles the antigen-specific immune response. While the impact of sleep has been best investigated for the consolidation phase of memory formation in the CNS and the immune system, the impact of sleep on the encoding and recall phases of immunological memory needs further investigation.

### C. Sleep Effects on Infection Outcome and Risk

Based on the finding that infections can increase and intensify NREM sleep, the hypothesis emerged that sleep is an important acute phase response of the body to help fight infections (544). An early study found that rabbits inoculated with different microbes not only exhibited increased SWS, but that the sleep response was an indicator of survival chance. Those animals exhibiting a stronger enhancement in NREM sleep amount and intensity had a more favorable prognosis and less severe clinical signs following infection with *Eschericia coli*, *Staphylococcus aureus*, and *Candida albicans* (546). Experimental studies manipulating sleep in animals support the notion that sleep affects the outcome of an infection. Kuo and Williams (311) showed that prolonging sleep duration using a genetic approach in *Drosophila* increased resistance to bacterial infection, as measured by bacterial load, and improved survival compared with flies with normal sleep duration. Interestingly, genetically reducing sleep duration did not affect the survival rate, but unexpectedly reduced bacterial load. This was interpreted as a reduced tolerance to infection, as the flies showed the same survival rate as control animals, even though they had a lower bacterial load. A reducing effect of sleep loss on bacterial load was also found after sleep deprivation for between 4 h and 3 days in normal flies (310, 582). The latter study also found an increased survival outcome following sleep deprivation, seemingly contradicting the findings from the same group using a genetic approach to manipulate sleep duration. A thorough analysis and series of experiments, however, revealed that the improved survival outcome was due to the rebound sleep that followed the sleep deprivation procedure, such that the sleep-deprived animals experienced enhanced sleep compared with the nondeprived group. Flies lacking two transcription factors necessary for the development of rebound sleep did not show an improved survival rate to bacterial infection following sleep deprivation (310). This supports the conclusion that it was not sleep deprivation per se that was responsible for the improved survival outcome, but rather the enhanced sleep following sleep deprivation (310). Together, these studies in flies indicate a positive effect of sleep on the outcome of a bacterial infection. Consistent with this conclusion, one study in mice found an increased mortality after experimentally induced sepsis in mice that underwent sleep interruption compared with a control group with undisturbed sleep (202). Studies in rats have shown that sleep deprivation for several days can even induce a translocation of commensal bacteria, which are harmless under normal conditions, into several extra-intestinal sites (41, 175). This translocation of bacteria has been postulated to eventually lead to general sepsis and death if sleep deprivation is prolonged (175). Hence, sleep is not only important for fighting infections induced by invading bacteria, but is also essential to keep in check usually harmless bacteria that naturally inhabit our body.

Recent studies have also investigated the impact of sleep on parasitic infection, including protozoa and helminths. Sleep deprivation for 72 h did not affect the number of intestinal worms in *Trichinella spiralis*-infected rats or the survival rate of mice after infection with *Trypanozoma cruzi* (249, 482). However, sleep deprivation before malaria infection increased the death rate and number of infected cells in mice, an effect that was reversed after animals had experienced rebound sleep (345).

Studies in humans have focused on the association between sleep and infection risk rather than infection outcome. Self-reported short habitual sleep (≤5 h per night) was associated with an increased risk for the development of pneumonia within the next 2 years (428) and with a higher incidence of reporting a respiratory infection within the past month (449) compared with 7–8 h sleepers. Interestingly, participants reporting a habitual sleep duration of ≤5 h per night, but indicating that they feel their sleep duration is adequate, did not have an increased pneumonia risk (428). This finding suggests that there are inter-individual differences in sleep needs, and that a short sleep duration can be sufficient for optimal immune function in people with lower sleep needs. This notion is in line with a study showing that participants reporting a perceived reduced immunity had poorer ratings of sleep quality, but did not differ in sleep duration compared with participants reporting a normal immune status (154). Using an experimental infection, Cohen et al. (119) showed that people reporting a short sleep duration in the weeks before they received nasal drops containing a rhinovirus had an increased risk of developing a clinical cold. This finding was replicated in a study in which sleep duration was measured objectively for 7 days using actigraphy (448). Another study measuring sleep with actigraphy in adolescents showed that short sleep duration was associated with a higher number of reported illness events, which included symptoms of cold, flu, and gastroenteritis (416). In contrast to these consistent findings, a recent study found no association between self-reported sleep duration and quality and the incidence of self-reported upper-respiratory tract infections (208).

Interestingly, the study by Patel et al. (428) reporting an association between short sleep duration and pneumonia risk revealed that participants sleeping 9 or more hours also had an increased infection risk. This finding is interesting in light of epidemiological studies demonstrating an enhanced mortality not only in short, but also in long sleepers (e.g., Refs. 22, 88, 235, 340). However, longer sleep may not be directly linked to increased mortality; it is thought that confounding variables such as (subclinical) disease mediate this association (22, 88, 340). The other studies mentioned above either did not investigate the association between long sleep and infection risk or did not find such a relationship (119, 448, 449). It therefore would be too early to draw any conclusions about the role of long sleep duration in infection risk.

In sum, the literature on short sleep duration and infection risk in humans convincingly demonstrates an association between both variables (although these studies did not manipulate sleep), and experimental studies in animals have proven causality for the role of sleep in infection outcome. Taking an evolutionary approach, Preston et al. (453) revealed that evolutionary increases in mammalian sleep duration are associated with a higher number of circulating immune cells and reduced levels of parasitic infections (encompassing viral, bacterial, and macroparasitic infections). These findings point to an important role of sleep for optimal immune defense and may explain why sleep survived evolution, despite being a potentially dangerous state of prolonged unconsciousness. Several aspects of the influence of sleep on the immune system remain to be explored, including how the sleep-induced changes in single immune parameters described in section III*A* relate to the positive influence of sleep on vaccination responses and infection. However, the various studies described in this and the previous section clearly indicate that sleep’s effect on immune parameters does indeed have functional relevance and is critical for optimal immune competence.

## IV. IMMUNE SYSTEM CHANGES ASSOCIATED WITH CHRONIC SLEEP DEFICIENCY

Over the last decade, a broad spectrum of disorders characterized by low-grade inflammatory upregulation has been identified, including neurological and neuropsychiatric disorders, degenerative diseases, cardiovascular disorders, metabolic disorders, and chronic pain conditions (100, 153, 337). The initiating trigger of inflammatory upregulation in these disorders is not well defined (377) (see also sect. I*C*). However, substantial evidence (mentioned in sect. III*A*) has accumulated showing that sleep deficiency could be a potent risk factor. In the following two sections, we will review studies on the association of immune measures with chronic sleep deficiency in the form of habitual short sleep duration and chronic sleep disturbances in population-based studies, including reports on insomnia disorder. Potential mechanisms through which chronic forms of sleep deficiency may lead to a chronic state of low-grade inflammation will be discussed in section IV*C*. Studies investigating the association between sleep duration and infection risk are discussed in section III*C* and are therefore not reviewed in this section.

### A. Immune Measures Associated With Habitual Sleep Duration

There is ample evidence that a short habitual sleep duration in population-based studies is related to adverse health outcomes (i.e., obesity, diabetes, cardiovascular disease, neuropsychiatric symptoms, pain) as well as mortality risk (for review, see Refs. 212, 573). A recent meta-analytic investigation of 40 prospective cohort studies enrolling over two million participants reported that shorter sleep duration (<7 h) in women and longer sleep duration (>8 h) in both women and men were associated with increased all-cause mortality risk (340). The relationship between longer sleep duration and mortality, while unexpected, has been reported in previous reviews and meta-analyses (88, 205, 214). It is thought to be due to residual confounders, such as uncontrolled comorbidities (429), illness-related longer sleep duration in the last months of life (537), among many other factors (313).

Inflammatory dysregulation has been suggested as a potential mechanism linking sleep duration and mortality, given that inflammatory markers appear to be independent predictors of mortality risk in older populations (78, 567). However, to date, only two investigations exist in this area. In a prospective cohort study including over 3,000 older adults followed over a 9-yr period, inflammatory markers (levels of IL-6, TNF, and CRP), lifestyle, and health status factors explained the association between higher mortality risk and self-reported short sleep duration (as indicated by an attenuated mortality hazard ratio in the model adjusted for these variables); the association with long sleep duration was predominantly explained by lifestyle and health status factors, but less so by inflammatory markers (228). Findings from a study in over 2,500 older men followed over a 7-yr period also suggest an inflammatory mechanism underlying the sleep duration-mortality relationship (515). Critically, this study assessed sleep objectively in the home environment through at-home actigraphy, recorded over a period of at least 5 days, in addition to a single-night PSG. Blood was collected fasted at a standardized time in the morning, thus controlling for inflammatory variations due to time of day effects and fasting status. Short sleep duration of <5 h was related to an increase in all-cause mortality risk through elevated inflammatory burden (i.e., composite measure of levels of CRP, IL-6, TNF, sTNF-RII, IFN-γ), as evidenced by a substantial attenuation of this relationship when adjusted for inflammatory burden. The inflammatory mediation appeared to be specific to short sleep duration, since the association between other sleep characteristics (e.g., sleep latency or time awake at night) was independent of the inflammatory burden. On a related note, elevated CRP has been well established as a clinically useful marker for cardiovascular risk prediction (470) and, therefore, may not only underlie the association between deficient sleep and mortality, but also the association between deficient sleep and increased risk for cardiovascular diseases (213).

A number of population-based studies have investigated the association between habitual sleep duration and inflammatory status independent of their mediating role in mortality and other health risks. In most of those studies, inflammatory status was measured by CRP or IL-6 levels in the systemic circulation, with some studies also assessing IL-1, IL-2, IL-4, IL-5 IL-10, TNF, IFN-γ, fibrinogen, adhesion molecules (e.g., sICAM, E-selectin), WBC counts and subsets, T cell subsets, and cell function. Sleep duration has generally been assessed by self-report through a single question (e.g., how many hours of sleep do you usually get at night during a normal work week?), with very few studies utilizing objective sleep assessment methods (e.g., PSG, actigraphy). Collectively, findings are variable: with respect to cytokines and other inflammatory proteins (for recent reviews, see Refs. 259, 511), some studies reported an association of these markers with short sleep duration, generally defined as <5 or 6 h of sleep compared with a reference range of 7–8 h of sleep (92, 105, 186, 367, 384, 430). Based on the longitudinal Whitehall II study in over 5,000 middle-aged adults, higher levels of CRP and IL-6 were not only associated with shorter sleep duration, but also with a reduction of sleep duration assessed 5 yr apart (186). A number of studies, however, also reported an association between elevated inflammatory markers and long sleep duration, defined as more than 8 or 9 h of sleep per night (92, 114, 156, 430, 452). Although associations between long sleep duration and elevated inflammatory status have been statistically adjusted for a number of factors (including demographic variables, health behaviors, medication, and medical comorbidities), uncontrolled disease processes are still thought to mediate this relationship. Of note, several studies did not find an association between inflammatory markers and sleep duration (268, 373, 450, 529, 533), adding to the heterogeneity of findings.

While most studies have focused on the assessment of cytokines and inflammatory proteins as indicators of inflammatory status, a few studies have examined immune cell counts, cell function/activity, and cell telomere length in the context of habitual sleep duration. A study in over 600 adolescents reported an association between high WBC counts and short self-reported sleep duration (<8 h/night) (136). Likewise, in another study in adolescents (*n* = 933), short sleep duration (<8 h/night) was associated with higher cell counts of total WBC, neutrophils, and monocytes, as well as higher counts of total T cells, CD4 T cells, memory T cells (CD3+RO+), and memory CD4 T cells (CD4+RO+) (435a). A reduction in naive T cell numbers was recently reported to be related to short sleep (<6 h/night) in a study with over 2,000 female participants (91). Such a decline in naive T cells may negatively affect the early immune response to novel antigens, and may underlie findings of a decreased antibody response after vaccination in short sleepers (447)(see also sect. III*B*). With respect to cell functions, which are generally assessed by the proliferation response of cells induced by antigens or mitogens, self-reported short sleep duration (<7 h) the night before blood collection in healthy adults was related to higher T cell proliferation in response to phytohemagglutinin as well as to lower NK-cell activity (192). In accordance with the latter findings, a decrease in NK-cell activity across two measuring time points has been found to be associated with a decrease in self-reported sleep duration in healthy women (504). These findings complement results of experimental studies showing a role of sleep in the regulation of lymphocyte proliferation and NK-cell activity (see sect. III*A3*). Recent studies linked sleep duration to immune cell telomere length, which is considered a measure of cellular aging (106, 284). In more than 400 healthy, middle to early old age participants drawn from the Whitehall II Cohort study, self-reported short sleep duration in men, but not in women, was related to shorter leukocyte telomere length (267). A similar association was found in older, but not middle-aged, adults (*n* = 154) (125) and was recently also reported in children (*n* = 1,567) (271). Ongoing inflammation has been shown to contribute to telomere dysfunction, although the exact interactions between inflammatory processes, including NF-κB activity and PRR activation, with telomere functioning are still not well understood and are currently under investigation (276). It is thought that shorter telomere length may reflect a mechanism or indicate a biological pathway by which shorter sleep duration is linked to long-term negative health consequences. However, research in this field is still relatively new, and future investigations are needed to further insights of the relation between sleep duration and processes of cellular senescence. The variability of findings reported for the relation of sleep duration to inflammatory cytokines, as reviewed earlier, is also true for the relation to immune cell counts, cell function, and telomere length. Of note, far fewer studies measuring these latter parameters have been conducted, and in some of those studies, habitual short sleep duration has been found to be unrelated to WBC counts (156, 405), NK-cell activity (391), and telomere length (451).

Inconsistencies in study findings are not surprising given the methodological limitations specific to population-based studies as well as methodological differences across studies. While population-based studies can give an estimate of the inflammatory consequences or correlates of habitual sleep duration, thus providing critical information beyond the effects of acute or subchronic experimentally induced short sleep duration in the laboratory setting, experimental studies can manipulate sleep in the controlled environment, thus helping to get insights into the directionality of the sleep-inflammatory relationship.

A major limitation of almost all of these population-based studies is the lack of a refined and objective sleep assessment methodology. Generally, sleep duration is obtained once by a single question; other sleep characteristics, including circadian misaligned or mistimed sleep (14, 589), and common sleep disorders such as apnea (140) or insomnia (191, 565) are generally not assessed, although they can substantially alter inflammatory status, in addition to habitual sleep duration. Furthermore, objective assessment methods (i.e., actigraphy, PSG) are rarely used. However, sleep duration evaluated by self-report questionnaires and diaries may not agree with the results of actigraphy and PSG. In healthy populations, self-reported sleep duration appears to be longer compared with actigraphy estimates, while in populations with sleep disturbances, self-reported sleep duration tends to be shorter (327, 375). In addition, the length of the sleep assessment period matters, and single day sleep assessments do not accurately reflect habitual sleep behavior (293). Depending on methods used for the assessment of sleep duration, findings on its relation to inflammatory markers may vary. For example, in the same study, sleep duration assessed by self-report correlated with levels of CRP and IL-6, while sleep duration assessed objectively by PSG correlated inversely with levels of TNF (430). Another methodological concern contributing to the heterogeneity of findings within and across studies is the lack of standardized blood collection times. The production of many inflammatory markers varies across the 24-h day (95). Unfortunately, blood collections are frequently not time-stamped; thus this information cannot be utilized for adjustment in analyses. Future population-based studies should therefore implement objective, multiple-day long sleep assessment methods to gain an accurate estimate of habitual sleep duration, and time-stamped blood collections to control for diurnal variability of inflammatory markers.

In summary, many, but not all, studies investigating habitual short sleep duration support an association with alterations in immune measures, including increased inflammatory proteins (in particular CRP, IL-6), increased WBC counts, reduced NK cell activity, and shortened telomere length. These alterations may contribute to increased mortality and disease risk reported for habitual short sleepers, and their potential causal role will need to be addressed in future studies.

### B. Immune Measures Associated With Chronic Sleep Disturbances

The use of the term *sleep disturbance* ranges from the presence of a single sleep complaint (e.g., difficulty falling asleep or frequent nighttime awakenings) up to the presence of insomnia disorder, in which a combination of sleep complaints is present for a certain time. In the general population, the one-year incidence rate is ~30% for sleep complaints and 7% for new-onset insomnia disorder (331). Despite high incidence rates of sleep disturbances, the immunological consequences and how they translate into increased disease risk are still not well understood. A recent meta-analytic review concluded that sleep disturbances show a stronger association with inflammatory upregulation (i.e., CRP and IL-6) than short sleep duration (259), supporting the importance of a better understanding of the inflammatory consequences of disturbed sleep. This section will first review the association between immune markers and sleep disturbances in population- and field-based studies, followed by a review of studies addressing the association in individuals suffering from insomnia disorder. Immune changes as they occur in breathing-related disorders (e.g., obstructive sleep apnea) are not discussed, as they are strongly related with hypoxia (72, 294), making it difficult to isolate the contribution of sleep disturbances to immune changes observed in this context.

#### 1. Population-based studies

Similar to population-based studies focusing on sleep duration, studies on sleep disturbance have most frequently examined cytokines and other inflammatory proteins (in particular CRP and IL-6, reviewed in Ref. 259), with a few studies examining immune cell counts or functions. Assessments of sleep disturbances are generally based on subjective judgement of sleep as good or poor using a graded scale, ratings of single or multiple sleep complaints (e.g., difficulty initiating sleep, difficulty maintaining sleep, nonrestorative sleep), or sleep questionnaires, such as the PSQI (87). If assessed objectively through PSG or actigraphy, sleep efficiency and the amount of WASO have been the most frequently used as indicators of sleep disturbances.

With respect to the acute phase protein CRP, a longitudinal prediction of CRP levels by sleep disturbances in a general population was first demonstrated in a study including nearly 3,000 young adults (108). Using cross-sectional study designs, a study in more than 8,500 participants showed that self-reported complaints of difficulty initiating sleep and feelings of nonrestorative sleep were associated with CRP levels in men only (328). A sex-specific association was also found in a cohort of more than 4,000 participants, where self-reported indices of sleep disturbances were related to higher CRP levels among men only (341). Other studies reported such an association in women only (268, 339, 445, 529). Furthermore, insomnia symptoms in combination with objectively-verified sleep duration of <7 h were associated with higher CRP levels as early as adolescence (*n* = 378) (183). A number of smaller scale studies (<200 participants) also support an association between self-reported indices of sleep disturbances and CRP levels (373, 408). The sleep disturbance-CRP relationship in the above-mentioned studies remained significant even after adjusting for several interfering factors, including age, smoking, obesity, cardiovascular risk factors, and depression. However, some studies reported a loss of association after adjustment for interfering factors (602), or did not detect a significant association between indices of sleep disturbances and CRP (156, 231, 367, 452).

With respect to inflammatory cytokines, a longitudinal study including nearly 3,000 participants reported that sleep disturbances (indexed by 6 components, such as sleep onset and sleep maintenance problems) predicted IL-6 levels measured 5 yr later (108). A cross-sectional study in fewer than 200 individuals found an association between poorer subjective sleep quality (assessed by the PSQI) and higher stimulated IL-1, TNF, and IL-6 production, of which only the association with IL-1 remained significant after adjustment for demographic and lifestyle factors (450). As with CRP, sex-specific associations have been demonstrated in a number of studies, including a longitudinal study in over 600 adults, in which lower subjective sleep quality at baseline (assessed with the PSQI) was prospectively associated with 5-yr increases in IL-6 levels in females, but not males (445). In accord with this finding, in another study only women showed an association between greater difficulty falling asleep and frequency of sleep disturbances (both PSQI subcomponents) with higher levels of IL-6 (529). On the basis of objectively assessed sleep, lower sleep efficiency was shown to predict higher IL-6 levels in 74 older women (201). In a sample of 254 adult men recruited from a manufacturing company, self-reported insomnia (defined as the perception of poor sleep combined with either difficulty falling asleep, difficulty maintaining sleep, or waking up too early one or more times during the past year) was associated with lower stimulated IFN-γ production and a lower IFN-γ/IL-4 ratio in the absence of other medical disorders (484). This suggests that self-reported insomnia appears to result in a shift towards Th2 immunity. Such a shift in the Th1/Th2 balance has been also observed for alcohol dependence-associated sleep changes (462) and in response to experimental sleep deficiency (24, 320), and may explain lower vaccine efficacy and increased infection risk in the context of sleep deficiency (see sect. III, *B* and *C*). Of note, several studies did not find an association between cytokines (TNF, IL-6) and sleep disturbances (231, 408, 452).

Beyond examining inflammatory markers under basal or resting conditions, very few studies have focused on inflammatory reactivity to a stressful challenge. Inadequate reactivity to physiological or psychological challenges likely contributes to increased disease vulnerability (reviewed in Ref. 374), and while reactivity to such challenges has been well studied for the classical stress systems (i.e., HPA axis and sympathetic nervous system), less is known about inflammatory reactivity, although inflammatory markers are reliably activated by stressful challenges (475). Accordingly, inflammatory reactivity could potentially be altered by sleep disturbances (82, 379). Supporting this hypothesis, poorer sleep quality was recently found to be associated with greater IL-6 reactivity following a psychosocial stressor in postmenopausal women (445). Furthermore, another study found that IL-6 reactivity following a series of cognitive challenges was higher in men and postmenopausal women age 50 and older who reported poor sleep quality, compared with those reporting good sleep quality (233).

With respect to immune cells, increased WBC counts in a sample of over 200 male workers in a manufacturing plant were associated with self-reported poor sleep (assessed by a single question), but not with sleep duration or shift work patterns, suggesting a critical role of the perception of sleep as poor in WBC upregulation (403). Based on objective sleep assessment through actigraphy, elevated WBC counts in a sample of almost 1,100 elderly individuals were associated with lower sleep efficiency and higher nocturnal wake time in women only; this association held even after controlling for various confounders, such as age, obesity, and hypertension (405). Furthermore, advanced epigenetic age and higher counts of late differentiated CD8 T cells, which are indicators of age-related changes in blood cell composition and immune senescence, correlated with insomnia symptoms in a study with nearly 2,100 women. Among insomnia symptoms, waking up at night was the most strongly related to older epigenetic age (91).

In addition, shorter leukocyte telomere length, a proposed marker of cellular senescence, has been linked with markers of sleep disturbances (assessed by the PSQI) in a sample of 245 healthy midlife women (451) and of 87 obese adults (446), with the latter study reporting a link with telomere length of CD8 and CD4 T cells in particular. Among older (but not middle-aged) adults from a healthy, small-scale (<200 individuals) community sample, good sleep quality (assessed by the PSQI) appeared to have a protective effect on age-related shortening of PBMC telomere length, as indicated by an attenuation of the age-telomere length association by better sleep quality (125). Such an age-specific association has been also observed in another study, where only the oldest age group (>70 yr) had shorter PBMC telomere length when diagnostic criteria of insomnia were fulfilled (90).

The link between sleep disturbances and immune measures has been also investigated in depression (124, 227, 393, 532), schizophrenia (176), alcohol dependence (462), hemodialysis (107, 458), organ transplantation (193), pregnancy (55, 409), cervical cancer risk (491), and diabetes (548), with most studies reporting a significant association between some aspects of sleep disturbances and immune measures.

Collectively, findings support a link between indices of self-reported sleep disturbances as observed in general/clinical populations and dysregulation of inflammatory markers, immune cell counts, and cellular aging markers. Similar to habitual short sleep duration, this suggests that these dysregulations may constitute a potential mechanism through which sleep disturbances influence disease risk.

#### 2. Insomnia disorder

Insomnia disorder is diagnosed if symptoms of difficulty initiating sleep, disrupted sleep, or waking up too early, combined with an impairment in daytime functioning, are present. Depending on diagnostic guidelines, insomnia symptoms must occur with a specific frequency (e.g., at least 3 times per week) and persist for a certain time (e.g., at least 3 mo). While the diagnosis is based on subjective reporting, a recent meta-analysis showed that the objective PSG profile in individuals with primary insomnia (i.e., insomnia not attributable to another disorder or substance abuse) is characterized by reduced total sleep time, longer sleep latency, increased number of nocturnal awakenings per night, and increased wake time after sleep onset, resulting in a decreased sleep efficiency compared with healthy controls. In addition, the amount of SWS and REM sleep appears to be slightly, but significantly reduced (25). Such PSG-derived sleep impairments are generally of a much lesser magnitude than subjectively reported sleep impairments. For example, total sleep time based on diary-based assessment is estimated to be 50 min less compared with PSG-based assessment (25).

There is good evidence that insomnia increases the risk for certain disorders including depression (439), hypertension (31), and type 2 diabetes (561). Dysregulations of immune markers have been suggested as a potential mechanism by which insomnia increases disease risk. However, while several studies (reviewed below) have pointed to immune dysregulations in insomnia disorder, no investigations have yet addressed their mediating role in the association between insomnia disorder and disease vulnerability.

In one of the first studies on immune system markers in insomnia disorder, a shift of the peak levels of systemic IL-6 and TNF from nighttime to daytime was observed in 11 individuals with insomnia without concomitant mental disorders, compared with healthy age- and body-mass index-matched control participants (565). The authors suggested that these daytime increases in inflammatory cytokines may explain increased fatigue experienced during the day. Of note, eligibility criteria for insomnia in this study included an objective criterion of <80% sleep efficiency during the screening PSG night. In contrast, another study reported an increase of IL-6 levels during the nighttime, rather than daytime, in 11 individuals with primary insomnia compared with age- and sex-matched healthy sleepers (84). This insomnia sample also showed objective PSG-derived sleep impairments, as indicated by increased sleep onset latency, reduced total sleep time, and decreased sleep efficiency compared with healthy sleepers. Furthermore, greater PSG-derived nocturnal wake time was related to higher IL-6 levels (84). A higher inflammatory composite score (IL-6, CRP, and monocyte counts) was further reported in 29 individuals with chronic insomnia disorder who were otherwise healthy [based on Diagnostic and Statistical Manual of Mental Disorders (DSM)-V criteria (20)] compared with age- and sex-matched control sleepers (191). This inflammatory effect was accompanied by objective sleep impairments, as indicated by a decrease in sleep duration (as measured by actigraphy) of 45 min daily in those suffering from insomnia disorder. In another study, counts of lymphocyte subpopulations (i.e., total T cells, CD4 T cells, and CD8 T cells) were found to be lower in 19 chronic primary insomnia patients (based on DSM-IV criteria) when compared with healthy sleepers, but no differences were observed for total leukocyte counts, NK-cell activity, or production of IL-1, IL-2, and IFN-γ (490). Of note, in the same study, individuals with insomnia and healthy sleepers also did not differ with respect to PSG-based sleep parameters (490), contrasting with subjective reports of higher nocturnal wake time and lower sleep efficiency in those with insomnia. Although speculative, this lack of difference in objective sleep parameters might explain the lack of difference in several immune parameters between insomnia patients and healthy controls in this study. To date, a single study has investigated telomere length in insomnia: In the oldest age group (>70 yr) within a sample of 126 older adults, a diagnosis of primary insomnia (based on DSM-IV criteria) was associated with shorter PBMC telomere length, suggesting that the presence of insomnia may accelerate cellular aging in the later years of life (90). Sleep was not objectively assessed in this study. Such abnormalities reported for basal immune and inflammatory markers in insomnia disorder may underlie inadequate influenza vaccine responses as recently reported (536). In this study, the vaccination response to influenza did not differ between 133 healthy college students with or without insomnia (based on DSM-V criteria), although the insomnia group had overall lower antibody amounts pre- and post-vaccination. When controlling for potential covariates, subjective sleep quality (assessed by the PSQI) and insomnia status were predictive of the influenza antibody response, which led the authors to suggest that both may attenuate immunity to the influenza virus (536). This complements findings of weaker vaccination responses in healthy participants with a short sleep duration (see sect. III*B*).

Collectively, findings suggest that a diagnosis of insomnia is associated with immune dysregulations, ranging from increased levels and diurnal rhythm changes of inflammatory cytokines to decreased lymphocyte subsets to shorter telomere length, and may potentially attenuate influenza antibody responses. Given that most studies excluded individuals with comorbid psychiatric and other disorders, many of which are known to affect both sleep and the immune system, findings strongly suggest that the presence of insomnia itself is directly linked to immune and inflammatory dysregulations. Furthermore, the magnitude of such dysregulations may depend on the presence of objective sleep impairments, which have been suggested as a marker indicating the biological severity and the medical impact of the disorder (184, 561).

To conclude, habitual short sleep duration and chronic sleep disturbances are related to a variety of diseases, including cardiovascular diseases, metabolic diseases, chronic pain conditions, some forms of cancer, and neuropsychiatric disease (255), all of which involve immune dysregulations. While such dysregulations have been found to be associated with indicators of short or disturbed sleep in population-based studies and with the diagnosis of insomnia in small-scale, well-controlled studies, it is still an open question whether they function as mechanisms underlying increased disease vulnerability in those suffering from short or disturbed sleep.

### C. Potential Mechanisms of Low-Grade Inflammation Associated With Sleep Deficiency

Sleep deficiency can induce low-grade inflammation, as indicated by increases in leukocyte counts and pro-inflammatory cytokines following experimental sleep deprivation or restriction (see sect. III*A*) or in the context of habitual short or disturbed sleep (see sect. IV, *A* and *B*). In section II*B3*, we elaborated on the possibility that environmental interactions can initiate an inflammatory response through PAMPs and DAMPs, which activate PRRs on immune and nonimmune cells. A simple explanation of how sleep loss might promote low-grade inflammation could be that any additional time spent awake enhances environmental interaction, potentially leading to activation of PRRs by PAMPs and DAMPs that will trigger inflammatory processes. In addition, SWS-dependent processes might actively counteract inflammation in the brain (64, 65, 302). Supporting this hypothesis, a study in healthy humans found that CSF levels of amyloid beta, a DAMP (115) that is released during synaptic firing, showed a rhythm parallel to the sleep-wake cycle (i.e., amyloid beta increases during the day and decreases overnight) (280). Furthermore, amyloid beta was found to be lower following a night of regular sleep compared with a night of total sleep deprivation (410, 509) or SWS disruption (278). It is assumed that the glymphatic system, a waste clearance pathway to eliminate soluble proteins and metabolites from the CNS, flushes amyloid beta away from the brain parenchyma during sleep (60, 81). As shown in animals, this system allows the drainage of deep brain structures resulting from an increase of cortical interstitial space, which expands by more than 60% during sleep (593). Disturbances in this metabolic clearance due to the lack of sleep can lead to neuroinflammation and, in the long term, to neurodegenerative diseases (60, 399). A parallel active clearance of IL-1 and TNF from the brain during NREM sleep has been suggested (116), but as yet awaits experimental evidence. In the periphery, recumbency and sleep-associated changes in the vasculature could likewise affect the draining of proteins from tissues and organs by the lymphatic system (143, 167, 168, 312). Both the glymphatic and the lymphatic systems eventually redirect proteins including sleep regulatory substances into lymph nodes and then into the circulation for final clearance by the liver or the kidneys (144, 145, 343). The role of sleep in these processes, however, is completely obscure. In rats, sustained sleep deprivation induced oxidative stress as well as DNA, cell, and tissue damage (173), likely representing a condition of increased DAMP levels and DAMP-driven inflammation. In this study, cell injury was most pronounced in the intestine (173), which could result in increased intestinal permeability (leaky gut) and, together with the impairment of host defense following sleep loss, to a translocation of commensal bacteria and PAMP-driven systemic inflammation (175, 303) (see also sect. III*C*). Whether sleep loss contributes in this way to intestinal dysbiosis and, consequently, to low-grade inflammation is still unclear (12, 37, 425, 444, 603). Finally, the HPA axis, which is crucial in suppressing inflammatory processes, might become impaired by (prolonged) sleep loss (379). Indeed, one study found that the interplay between inflammatory and stress markers at the cell level, as assessed in vitro by the sensitivity of monocytes to the counter-inflammatory cortisol signal, was dysregulated following prolonged sleep restriction in humans (512).

Notably, chronic sleep disturbances may be the cause or the consequence of other known triggers of low-grade inflammation, including circadian disruption (370), obesity (406), adverse lifestyle habits like physical inactivity (431) and unhealthy food (508), and psychosocial influences like stress (121, 189, 454, 525), loneliness (120), and low socioeconomic status (166). These are all factors that should be controlled for in studies linking short or disturbed sleep and inflammatory processes.

In sum, available evidence in animals and humans suggests that sleep counteracts low-grade systemic inflammation and in this way maintains immune homeostasis. In section II*D*, we outlined that chronic inflammatory conditions are associated with sleep disturbances, and it is conceivable that physiological sleep regulation fails when inflammatory mediators are constantly elevated. However, sleep disturbances in inflammatory conditions presumably also have a negative effect on the underlying immune process, thus feeding into a vicious circle.

## V. SLEEP TO REINSTATE IMMUNE BALANCE

While recovery sleep has been well studied with respect to the reversibility of neurobehavioral consequences of sleep deficiency, the question of whether immune and inflammatory consequences return to normal once recovery sleep has been obtained has only recently gained attention. Within the neurobehavioral domain, for example, various doses of recovery sleep (0, 2, 4, 6, 8, and 10 h) following five nights of experimental sleep restriction resulted in a sleep-dose-dependent decrease of lapses (i.e., reaction times over 500 ms) in the psychomotor vigilance test (28) (reviewed in Ref. 30). Furthermore, performance did not return to baseline levels after a single night with a 10-h sleep opportunity (28) or three nights with an 8-h sleep opportunity (32), suggesting that a longer recovery sleep period is needed to fully resolve any performance decrements due to experimental sleep deficiency. Although such elegant sleep-dose recovery responses have not yet been conducted for immune and inflammatory processes, the following sleep recovery options have been examined as potential countermeasures of the inflammatory consequences of sleep deficiency and will be reviewed below: *1*) recovery sleep following experimentally induced sleep deficiency, *2*) daytime napping, and *3*) extension of habitual sleep duration. Furthermore, recent studies investigated the immune effects of cognitive behavioral therapy (CBT) in chronic sleep disturbances, such as insomnia disorder. While the effects of recovery sleep have been studied in both experimental animal and human models, there are no animal models on the effects of nap behavior, sleep extension, or interventions equivalent to human CBT.

### A. Recovery Sleep Following Acute Sleep Deficiency

In humans, one of the first studies investigating immune recovery from total sleep deprivation (i.e., 64 h continuous wakefulness) in healthy adults reported that monocytes still tended to be increased after a single night of recovery sleep (duration not reported) compared with pre-deprivation levels, while recovery levels of leukocyte counts, granulocyte counts, and NK-cell activity returned to pre-deprivation levels (152). In a study using a total sleep deprivation protocol, increased numbers of leukocytes and monocytes assessed once in the morning following 2 days of continuous wakefulness in healthy young men normalized after a single night of recovery sleep compared with control sleepers (480). However, increases in CD4 T cells remained elevated even after three nights of recovery sleep (480). In a study utilizing frequent blood sampling over 51 h in healthy young men, recovery sleep following a single night of sleep deprivation fully restored the sleep deprivation-induced increase in monocyte counts (67). Nocturnal increases in lymphocyte counts, including NK cells, CD4 and CD8 T cells, and activated T cells, were reduced in the evening following sleep deprivation compared with a sleep control group. A further reduction of NK cells and activated T cells was observed during recovery sleep, indicating a compensatory decrease of those cell subsets (67). Furthermore, a single night of recovery sleep limited to 8 h following a single night of sleep restricted to 2 h was not able to recover increased WBC counts (mainly due to elevated neutrophil subsets) assessed at 7 AM in healthy men (178). However, when recovery sleep was extended to 10 h in healthy men, both WBC and neutrophil counts normalized. Monocyte counts, in contrast, were not affected by either sleep restriction or recovery sleep, while lymphocyte counts nonsignificantly decreased in response to sleep restriction, with a further significant decrease following 8 h of recovery sleep. Again, extending the recovery night to 10 h appeared to dampen the lymphocyte decrease observed after an 8-h recovery night to nonsignificance (178). These findings suggest that increasing the amount of recovery sleep obtained in a single night can help to normalize consequences of sleep deficiency on cell counts. Using an experimental model in which five nights of sleep restriction (4 h of sleep/night) were followed by two nights of recovery sleep (8 h of sleep/night), numbers of B lymphocytes, but not T lymphocytes, increased in response to sleep restriction, and were restored to normal values after the two recovery nights (556). A recent study investigating immune cell subpopulations in healthy young men employed a longer recovery period of 7 days (8 h of sleep/night) following 5 days of sleep restriction (4 h of sleep/night) (326). Counts of total WBC, monocytes, neutrophils, and lymphocytes gradually increased across the days of sleep restriction. While counts of monocytes and lymphocytes returned to baseline levels after a single night of recovery sleep, neutrophil counts were not restored to pre-restriction levels even after 7 days of recovery sleep (326). Furthermore, this is one of the few studies utilizing frequent blood sampling throughout the day and found a flattening of the diurnal rhythms of WBC and neutrophil numbers in response to sleep restriction, which appeared to be exaggerated at the end of the recovery period, as indicated by a higher amplitude of the diurnal curves (326). These findings suggest that restoration of not only the number, but also diurnal rhythms of some immune cell populations require prolonged periods of recovery sleep.

Besides recovery of circulating immune cells from sleep deficiency, a few studies investigated recovery of cytokines and inflammatory proteins. Spontaneous production of IL-6 and TNF by monocytes was increased in the morning after a night of sleep restriction, and this increase persisted in those monocytes that co-produced IL-6 and TNF after a night of recovery sleep (264). In another study, an increase of LPS-stimulated monocytes co-producing IL-6 and TNF after a night of sleep restriction persisted after a night of recovery sleep in younger (<40 yr), but not older participants (>60 yr) (89). Mimicking sleep patterns of a common week with 5 days of restricted sleep (4 h of sleep/night) followed by 2 days of recovery sleep (limited to 8 h of sleep/night), sleep restriction induced elevations of IL-1, IL-6, and IL-17 (as assessed at the mRNA level of stimulated PBMCs) (556). These parameters were still elevated, though nonsignificantly, after two nights of recovery sleep. Circulating CRP levels even continued to increase after recovery sleep (556). However, another study showed that when recovery sleep was extended to 10 h per night for three nights, elevated circulating IL-6 levels resulting from 6 days of sleep restriction (6 h of sleep/night) returned to normal in healthy adults (432). Such findings suggest that sleeping in on the weekend (i.e., extending habitual sleep duration) and/or having multiple days of recovery sleep may help to resolve inflammatory modifications due to acute sleep deficiency.

In recent years, the chronicity of common patterns of sleep restriction with intermittent recovery sleep has been experimentally modeled to examine processes of inflammatory adaptation to the repeated exposure to such patterns. A common feature of many biological systems is to habituate (i.e., to decrease in response) when repeatedly exposed to the same stressor or challenge (215). Whether this is also true for the repeated exposure to sleep deficiency is not known. Using an experimental model mimicking common patterns of sleep restriction during week days (4 h of sleep/night) and recovery sleep during weekend days (8 h of sleep/night) over a 3-wk-long period in healthy young adults, one study found that stimulated IL-6 expression by monocytes increased during the second and third week of sleep restriction and remained elevated above baseline levels after recovery sleep was obtained (512). These findings suggest that the inflammatory system does not habituate to the repeated exposure of common sleep restriction-recovery patterns over a 3-wk period. The inflammatory consequences of exposure to such patterns over a longer period of time (e.g., years or decades), as are often experienced in real life, still need to be addressed in future studies.

In animals, investigations on the effects of recovery sleep on immune parameters are rare. A recovery sleep period of 48 h reversed the reduction in survival of a malaria infection, induced by prolonged REM sleep deprivation in mice; in contrast, 24 h of recovery sleep were not enough to reverse the sleep-deprivation-induced effects (345). In rats, 48 h of recovery sleep following 10 days of sleep deprivation restored sleep deprivation-induced increases of myeloperoxidase activity (an enzyme constituent of neutrophils) in the lung and liver. However, myeloperoxidase activity in the intestines was increased after recovery sleep compared with baseline and sleep-deprivation levels, suggesting ongoing reactive processes during recovery sleep subsequent to sleep deprivation (174). In rats undergoing prolonged sleep restriction for 21 consecutive days, a decrease in total leukocyte counts normalized after 24 h of recovery sleep, but did not normalize after recovery sleep following a REM sleep deprivation protocol over 4 consecutive days (596). In light of a number of other immune measures modulated by these protocols (not reviewed), findings suggest that immune recovery depends on the type and duration of prior sleep loss, with considerable response variation between different immune measures.

In summary, while research on immune recovery is still in its early stages, current findings suggest that restoration of sleep deficiency-induced modifications of some (but not all) immune markers, as well as their diurnal rhythmicity, takes longer than a single or even a couple of nights of recovery sleep. Future studies are warranted to identify the amount of recovery sleep required to gain full immune and inflammatory restoration following exposure to sleep deficiency. In light of current research findings, recovery needs will likely differ for different immune and inflammatory markers, with some markers restoring quickly and others requiring prolonged sleep recovery periods involving extended sleep per night or multiple nights of recovery sleep; based on current findings, neutrophils and IL-6 likely fall into the latter category.

Future studies are also warranted to identify mechanisms contributing to slow or incomplete recovery of inflammatory markers. Counter-inflammatory pathways likely play an important role in establishing complete inflammatory recovery. Signals with counter-inflammatory properties include the classical stress hormone cortisol, anti-inflammatory cytokines (such as IL-10), and the newly discovered omega-3- and -6-derived inflammatory lipid mediators (such as resolvins and maresins), which actively resolve inflammation (502). A better understanding of the role of counter-inflammatory pathways may help to develop pharmacological strategies to ameliorate the inflammatory consequences of sleep deficiency, thereby reducing inflammatory-related disease risk.

### B. Daytime Napping

Like research on nighttime recovery sleep, research on the pro- and/or anti-inflammatory consequences of daytime nap behavior is still in its early stage. Within population-based studies, the effects of habitual napping on inflammatory markers appear to be complex. For example, in a study with over 300 participants, self-reported nappers had increased circulating CRP and IL-6 levels compared with non-nappers, but this relationship was influenced by a number of factors, including nap frequency, nighttime sleep duration and quality, and health status (142). Self-reported napping assessed in over 5,000 older participants was also associated with higher CRP levels, particularly in older participants, but the relationship was attenuated after adjusting for health-related variables such as pre-existing diseases, blood pressure, and depression (335). An association between increased napping and elevated CRP has been also found in a young population of over 2,100 adolescents (358). Again, complex interactions among daytime nap behavior, nighttime sleep, health status, and CRP levels were found. The directionality of the relationship between daytime napping and inflammatory markers remains unknown in these non-laboratory population-based studies, although it has been argued that nap behavior most likely results from underlying health problems (see also the review in Ref. 177).

In the controlled laboratory setting, a few studies have examined the effect of daytime napping as a counter-measure of the inflammatory effects of acute sleep deficiency. A mid-afternoon nap of 2 h has been shown to reverse the effects of one night of total sleep deprivation on circulating IL-6 levels in young healthy participants (563). Similarly, two 30-min-long naps (in the morning and afternoon, respectively) normalized daytime IL-6 decreases observed after a night of sleep restricted to 2 h (180). In another study, adding a short early afternoon nap of 30 min to a standard 8-h long sleep recovery night was sufficient to normalize increased neutrophil counts that resulted from prior exposure to a night of sleep restricted to 2 h (178).

Collectively, findings generated in the laboratory setting suggest that napping appears to be an effective counter-measure that supports the recovery of immune changes induced by acute sleep deficiency. Opposing findings from population-based studies, where napping appears to be associated with elevated, rather than decreased or normal inflammatory markers, are thought to reflect underlying health problems, a hypothesis that warrants future investigations. A better understanding of the influence of nap characteristics, such as nap frequency, duration, placement during the daytime, as well as interactions of daytime nap behavior with nighttime sleep behavior on inflammatory status will likely help to optimize nap behavior as a potential inflammatory countermeasure of sleep deficiency.

### C. Extending Habitual Sleep Duration

Considering that cutting back on sleep on a regular basis is a common behavior, extending habitual sleep duration may help reduce immune/inflammatory changes associated with habitual short sleep (i.e., sleep duration of ≤6 h/night). However, to date, only a few studies have explored inflammatory changes after sleep extension. In one study, participants with pre- or type 1 hypertension and habitual sleep duration of <7 h per night extended their bedtimes for 60 min every day over 6 wk, which resulted in an actigraphy-based increase in sleep duration of ~35 min per day (225). This increase of habitual sleep duration by ~10% per day resulted in a substantial decrease in blood pressure, while inflammatory markers (i.e., WBC counts and circulating levels of CRP and IL-6) nonsignificantly decreased from pre- to post-intervention. Given insufficient statistical power to detect inflammatory changes in this study, future studies with an adequate sample size need to substantiate these preliminary findings. A recent study in healthy young men increased bedtime by 1.5 h over 6 consecutive days (103). PSG-derived total sleep time between the extended bedtime condition (9 PM–7 AM) and habitual bedtime condition (10:30 PM–7 AM) differed by ~100 min on average, mainly due to increases of sleep stages 1, 2, and REM sleep in the extended bedtime condition. However, no differences were observed in lymphocyte, monocyte, or neutrophil numbers in the blood (103). Of note, blood was assessed only once per day, which may have reduced the chance of detecting transient changes in cell numbers. Furthermore, the amount of SWS, which may play a critical role in leukocyte subset changes (49), was not changed by the bedtime extension condition. In summary, research on the effects of sleep extension on immune parameters is still in its early stages, and further studies are needed to evaluate whether extending sleep duration is beneficial for the immune system.

### D. Cognitive Behavioral Therapy for Insomnia

CBT for insomnia (CBT-I) is considered the first-line treatment for insomnia (557) and outperforms pharmacological interventions, in particular with respect to maintenance of therapeutic effects at long-term follow-up (270, 513). In recent years, studies on CBT-I have incorporated a physiological measuring arm, including the assessment of immune parameters, which will be reviewed below. The impact of pharmacological sleep interventions, such as benzodiazepines (e.g., triazolam) and nonbenzodiazepines (e.g., zolpidem) (reviewed in Ref. 159) on immune parameters has received very little attention and is not discussed here. The interested reader is referred to recent reviews on the effects of pharmacological sleep therapeutics on specific immunological aspects (i.e., wound healing and infection risk) (163, 277).

In one of the largest CBT-I studies with over 100 older adults suffering from primary insomnia, CBT-I resulted in lower levels of CRP throughout the 16-mo follow-up period compared with adults receiving sleep education, and this decrease was associated with remission of insomnia (258). Furthermore, the percentages of monocytes producing TNF alone or in combination with IL-6 were lower 2 mo post-treatment (257), but not during later follow-up time points. Using a genome-wide transcriptional profiling approach, genes that were downregulated after CBT-I included transcripts involved in inflammation, while upregulated genes included transcripts involved in IFN and antibody responses (257). These immune effects suggest that CBT-I in adults suffering from insomnia lowers inflammatory risk. The impact of CBT-I on immune markers has also been investigated in medical populations with sleep disturbances. In women with insomnia disorder secondary to breast cancer, LPS-stimulated production of IL-1 and IFN in whole blood was increased from pre-to-post CBT-I treatment (492), although whether these changes were paralleled by CBT-I-induced sleep changes was not addressed. In peritoneal dialysis and hemodialysis patients, CBT-I improved sleep quality compared with a sleep hygiene education intervention, which was paralleled by a decrease of serum levels of inflammatory markers, including IL-1 (102), CRP, and IL-18 (101).

Interpretation of immune changes in response to CBT-I can be challenging, in particular in clinical populations where the underlying medical disorder potentially interferes with the immune status. However, overall, findings suggest that improving sleep disturbances has a beneficial effect on various immune parameters, which potentially may mediate the association between sleep disturbances and increased disease risk reported in epidemiological studies (see sect. IV*B*).

To conclude, recovery sleep following acute sleep deficiency, naps, extension of habitual sleep duration, and CBT-I may counteract sleep deficiency-induced changes of immune measures. Future research in this young field will need to investigate optimal strategies, for example, with respect to placement and length of recovery sleep/naps, to successfully restore sleep deficiency-induced immune changes.

## VI. CONCLUSIONS AND FUTURE DIRECTIONS

### A. Conclusions

#### 1. Immune-to-sleep directionality

Sleep and the immune system are bidirectionally related. Substances of the immune system, in particular the cytokines IL-1 and TNF, and PGD_2_ are involved in the regulation of spontaneous, physiological sleep in animals, and also mediate the increase of NREM sleep amount and intensity after the animal’s host defense is challenged with pathogens. These (and other) cytokines and PGs are potentially also involved in the physiological sleep regulation and pathogen-induced sleep responses in humans, although experimental evidence is still weak. Experimental studies on the application of pathogens and components of pathogens (e.g., endotoxins) in humans, as well as field studies in individuals with chronic infectious and inflammatory diseases, show that sleep changes are common, but can vary greatly, including enhanced, reduced, disrupted, or misplaced sleep. The type of sleep change likely reflects a combination of various factors, including the type of pathogen and severity of host defense responses. In the case of chronic infectious or inflammatory diseases, disease activity, stage, symptom severity, and comorbid disorders play a role, too. However, the immune system’s involvement in disease-associated sleep changes is probably best supported by recent research on the effects of immunotherapy (e.g., anti-TNF treatment) on sleep, which shows that dampening activity of certain inflammatory cytokines improves sleep quantity and quality. While it has been shown that PAMPs are upstream molecules of the increase in sleep-regulatory substances, the role of DAMPs in this context is still unknown.

#### 2. Sleep-to-immune directionality

##### A) EXPERIMENTAL STUDIES.

Several experimental studies in animals and humans show that manipulation of sleep can affect a wide array of immune parameters, including leukocyte migration and distribution, cytokine production, leukocyte activity and proliferation, antibody levels, complement activation, expression of cell adhesion molecules, and immune-related genes. It is therefore not surprising that sleep influences infection outcome, as well as the response to vaccination as an experimental model of infection. Studies in flies and rodents suggest that sleep increases the survival of an infection, whereas several studies in humans indicate that sleep loss increases the susceptibility to infections and worsens the immune response to vaccination. Mechanisms underlying the effect of sleep on the immune system are not well understood. The current literature supports the hypothesis that SWS, by inducing an immune-supportive hormonal constellation, fosters and coordinates the migration of T cells and APCs to lymphatic tissues as well as the communication between these cells. This may lead to an improved adaptive immune response and stable immunological memory formation. The effect of sleep on functional aspects of the innate immune response has been less thoroughly investigated.

##### B) FIELD STUDIES.

Findings of studies in the natural setting show that sleep duration (short and long) and sleep disturbances (including insomnia disorder) are linked to dysregulation of inflammatory markers, immune cell counts, and cellular aging markers. These immune dysregulations may constitute a potential mechanism through which sleep influences disease risk, which has been supported in more recent studies. However, there is a considerable variability between studies, particularly between those investigating immune correlates of habitual sleep duration. As outlined in this review, this likely stems from a number of methodological differences, particularly the lack of objective sleep assessment and standardized timing of specimen collection in these studies. Implementing objective, multiple-day-long sleep assessment methods (to gain an accurate estimate of habitual sleep duration) and time-stamped blood collections (to control for diurnal variability of inflammatory markers) may diminish the variability of study findings in the future.

Box 1. **Callout box for clinicians**

•  Sleep is a biological need, and adequate sleep duration and quality help maintain immune health.
•  Patients should be educated to attain sufficient sleep after receiving a vaccination to strengthen the vaccine response.
•  Adequate sleep duration can improve infection outcomes and is associated with reduced infectious disease risk.
•  Chronic sleep deficiency disturbs immune homeostasis, thereby presumably increasing the risk for the development and amplification of several diseases in which immune dysregulation is common (e.g., cardiovascular, metabolic, autoimmune, and neurodegenerative diseases).
•  Clinicians should be aware that many diseases are comorbid with sleep disturbances and encourage the patients to improve their sleep behavior and educate them about sleep hygiene (i.e., good sleep habits). This may have a beneficial effect on the severity and progression of the disease.
•  Various medications can disturb sleep (e.g., beta-blockers, corticosteroids, analgetics, anti-depressants), which needs to be considered in treatment decisions.

#### 3. Clinical implications of the sleep-immune crosstalk

In addition to infectious and chronic inflammatory conditions that involve a clear immunopathology (e.g., HIV, RA), numerous other diseases emerged in the last decades for which mild systemic elevations of inflammatory markers are characteristic, including cardiovascular (481) and metabolic diseases (153), certain forms of chronic pain (23), certain cancers (165), and neurodegenerative diseases (9). In all these diseases, deficient sleep is common (279). As we have outlined in this review, sleep deficiency induces immune system changes, and vice versa, the immune system modulates sleep. Consequently, targeting sleep will need to be an integral part of disease prevention and treatment strategies, while at the same time, research continues to address the knowledge gaps (see next section). Examples of clinical translations of knowledge gained in the sleep-immune field are outlined in the following (see also Box 1):

•  Given that sufficient sleep promotes an optimal vaccination response, individuals should be informed to have sufficient sleep when receiving a vaccination. Vaccinations will likely benefit also from wiser timing. Thus, depending on the type of antigen, the beneficial effects of sleep on vaccination outcomes may be more pronounced following vaccination either in the morning or in the evening.
•  The knowledge about the relationship between sleep and infection risk/outcome is most relevant for inpatients in hospitals, as infections are a serious risk factor for multi-morbid and immunocompromised patients. Sleep disruption is a well-studied and highly prevalent problem in intensive care units (e.g., Refs. 204, 440). Reducing factors responsible for sleep disturbances in the hospital environment such as exposure to constant noise and light are likely to minimize complications and improve recovery (440).
•  Sleep does not only affect immune functions related to infection, but also those to several other immune-related pathologies. For instance, sleep disturbances can increase allergic reactions and worsen tumor-related immune responses (138, 226, 289, 404), which indicates that patients with these diseases could profit from sleep improvements. In other situations, however, patients may profit from controlled, short-term sleep deprivation, in analogy to employing sleep deprivation as antidepressive treatment in mood disorders (128). Organ transplantation might be such a clinical condition that could take advantage of the immunosuppressive effects of sleep deprivation: One study reported a prolonged allograft survival after sleep deprivation, which was accompanied by a reduced number of graft-infiltrating CD4 T cells (479). However, much research will be needed to investigate whether there is an optimal “dosage” of controlled sleep deprivation in such specific contexts that outweighs the widespread negative consequences of sleep loss.
•  Postoperative pain is a major health care challenge that remains undermanaged (590). In human and animal models, sleep disturbances pre- and post-surgery have been shown to be associated with prolonged postoperative recovery time and increased pain (reviewed in Refs. 110, 229, 571), one of the cardinal signs of inflammation. Hence, pre- and post-operative sleep management will likely reduce postoperative pain and accelerate recovery processes.
•  A number of medications for the treatment of various diseases have sleep-disturbing effects, thereby potentially decreasing the immunosupportive effects of sleep. Sleep-disturbing or -altering effects have not only been shown for psychotropic medications (e.g., antidepressants), but also nonpsychotropic drugs, including cardiovascular drugs (e.g., beta-blocking agents), corticosteroids, and analgetics (e.g., opioids, NSAIDs) (reviewed in Ref. 554). Appropriate dosage and timing of medications or medication change should be considered to keep associated sleep-disturbing effects at a minimum.
•  In disorders characterized by immune dysregulations, immune-therapy may not only be used to improve disease activity, but also to directly improve sleep. As described in section II*D2*, treatment of RA patients with IL-6 or TNF blockers improved sleep (196, 283, 535). Comparable effects of anti-TNF therapy on sleep were found in patients with IBD and ankylosing spondylitis (282, 527). Along a similar line, administration of a TNF antagonist to abstinent alcohol-dependent patients counteracted increases in REM sleep, which are prospectively associated with the risk of alcoholic relapse (260).

### B. Future Directions

#### 1. Variability in methods influence the outcome of experimental studies

As mentioned in the previous sections, several factors influence the sleep-immune relationship (FIGURE 1). The methods of manipulating sleep vary greatly, especially in animal studies. Some methods induce greater amounts of stress, as defined by HPA axis and sympathetic nervous system activation, which can affect immune parameters independently of the sleep manipulation (423, 530). Therefore, methods keeping stress factors to a minimum or appropriately controlling for them should be used in future animal studies. For short-term sleep deprivation, gentle handling techniques (see TABLE 1) may be considered the methods of choice as they avoid possible stress effects associated with movement restriction, forced activity, and contact with water, which are inherent to other methods (8, 476).

Sleep can be deprived totally (no sleep allowed during the entire manipulation period) or partially (altering periods of forced wakefulness with opportunities for recovery sleep). Other methods induce disruptions of sleep continuity or selectively deprive only single sleep stages. The duration of sleep manipulation also varies greatly between studies, from only a couple of hours to several weeks. This can lead to different, and sometimes opposite, outcomes, as the body may try to compensate for the effects of sleep loss. Environmental factors, such as light exposure, ambient temperature, body posture, food, and fluid intake, have been controlled to different degrees in different studies. As described in previous sections, the type of immune assay is also relevant when assessing the outcome of sleep manipulation. Finally, sleep-immune interactions might also vary between different species.

Taken together, all these factors can greatly influence observed outcomes and might explain divergent results from different studies. Therefore, it is essential that future studies control for such factors and consider that different manipulation techniques, species, and employed assays can influence findings. In addition, only recently, approaches that enhance sleep (e.g., sleep extension, acoustic stimulation, long-sleeping fly mutants, see also TABLE 1) are becoming more frequent. These methods are an important addition to the sleep deprivation studies as they allow investigating the active role of sleep (rather than the effect of a lack of sleep). We therefore encourage employing such methods more frequently in future studies.

#### 2. Understanding the sleep-immune relationship across various time dimensions

Findings on the sleep-immune relationship also depend on a number of time dimensions, including time of day of parameter assessment or experimental intervention (e.g., morning, evening, night hours), age of the animal/participant (e.g., infancy, adolescence, older age), duration of infectious or inflammatory disease (e.g., early or late disease stages), and chronicity of sleep deficiency (e.g., acute, subchronic, chronic; FIGURE 1). Understanding the influence of these time dimensions on the relation between sleep and the immune system is valuable for developing pharmacological and behavioral strategies to support optimal immune and sleep health. However, knowledge in these areas is still limited.

With respect to time-of-day effects, this review showed that the effects of sleep and sleep loss on various immune parameters can vary considerably, partially due to differences between studies in the time and frequency of specimen sampling. Such time-of-day dependency is well-known for the stress hormone cortisol, where the effects of sleep loss result in increased, decreased, or unchanged levels, depending on time of day of measurement (see, for example, Refs. 27, 512). With exception of the major immune cell sets (e.g., leukocytes, monocytes), knowledge about diurnal variations of other immune parameters (particularly cytokines) and how they are affected by various challenges, including sleep modulation, is limited. Such knowledge may inform decisions in chronotherapy, which focuses on the optimal timing of the administration of (e.g., immunomodulatory) drugs to maximize therapeutic effectiveness.

Changes in the sleep-immune relationship across the lifespan are also poorly understood. Both sleep and the immune system undergo complex changes across the lifespan. For example, as the human body ages, both innate and adaptive responses functionally decline (357), a process termed immunosenescence (5). With respect to sleep, its duration shortens over the lifespan, and the amount and intensity of SWS starts to decline in adolescence (514). To date, experimental knowledge on the immune-sleep relationship is primarily derived from studying young adults (or young animals); generalizability to infants, children, adolescents, middle-age, and the elderly is limited. Understanding the immune-sleep relationship in age groups other than young adults may help answer important questions, such as the degree to which the observed age-related functional immune system decline is a consequence (or a cause) of sleep changes observed in the elderly. Again, such knowledge could potentially translate into targeting specific sleep components to prevent or ameliorate an age-dependent decline in immune functions.

Furthermore, when evaluating sleep and immune changes in chronic infectious or inflammatory diseases, the association depends on disease duration. The sleep-immune association may not be static over time, but may differ depending on the stage of a disease. This knowledge potentially benefits the optimization of therapeutic interventions at different stages of a disease.

The effects of the chronicity of sleep deficiency (i.e., the time span, from a few days to years or even decades, over which sleep remains deficient) are also under-studied. Understanding the adaptive processes that take place within the immune system in the course of acute to more chronic forms of sleep deficiency are likely essential in elucidating the mechanisms by which long-term sleep deficiency increases disease risk. Given that a key feature of most biological systems is the ability to adapt to ongoing adversity, the degree to which immune responses adapt to ongoing or chronic sleep loss warrants further research.

#### 3. Development of methods to assess physiological sleep need for optimal immune homeostasis

Currently, categorization of habitual sleep duration as short, optimal, or long generally relies on common recommendations (573) and/or self-perception. Self-perception, however, may not accurately reflect the sleep duration needed for optimal health maintenance. This has been suggested by research in neurobehavioral performance domains, where performance increasingly deteriorates with prolonged total sleep deprivation, but subjective sleepiness adapts to the sleep loss challenge. Thus, over time, the degree of performance decrement diverges from the perception of sleepiness. Such dissociation has been also shown for inflammatory markers, which remained increased in the course of ongoing sleep restriction, while subjective estimates of sleepiness tended to decrease over time (512). Thus self-reported sleep need may not accurately reflect sleep needed for optimal maintenance of biological systems, including the immune system. Current active research in the biomarker field will hopefully lead to the discovery of a marker that can measure inadequate sleep (316, 397), which may greatly assist in determining the optimal habitual sleep duration for maintaining immune homeostasis.

#### 4. Investigating the imbalance between pro- and counter-inflammatory signals

Research investigating the interaction between sleep and inflammation has mostly focused on the pro-inflammatory side, while the balance between pro- and counter-inflammatory signals, including anti-inflammatory cytokines (IL-10), pro-resolution lipid mediators (resolvins), and classical counter-inflammatory hormones (cortisol), has received little attention. Inflammatory balance is likely of key importance in assessing the interaction between sleep and the immune system. In addition, research examining the relationship between sleep and the inflammatory system generally investigated only a single or very few immune outcomes. However, the inflammatory response is regulated by a large number of pro- and counter-inflammatory mediators. Advances in profiling a large number of signals through the use of omic approaches may lead to a more complete understanding of the complexity of the sleep-immune relationship.

#### 5. Investigating sex differences in the sleep-immune crosstalk

Research on the relationship between sleep and the immune system has rarely addressed differences between females and males. Indeed, until recently, research in animals (and in some laboratories also in humans) has been mostly performed in males, to avoid potential confounding effects of the female estrogen cycle. However, sex differences exist in the prevalence of sleep disturbances as well as in the prevalence of various immune-related diseases or conditions: insomnia complaints are more common and severe in females than males (reviewed in Ref. 434), and this preponderance emerges already after late puberty (600). Autoimmune diseases (e.g., RA), chronic pain (e.g., fibromyalgia), and neurodegenerative diseases (e.g., Alzheimer’s disease) are more common in females than males and can also manifest and progress differently between sexes (reviewed in Refs. 185, 292). With respect to infectious diseases, females have a higher fatality of influenza A infection, a higher risk of developing AIDS, and stronger vaccine responses than males (reviewed in Ref. 292). To understand differential susceptibility to diseases and responses to vaccination between females and males, systematic investigation and reporting of sex differences with regard to the sleep-immune crosstalk are warranted.

#### 6. The future of sleep-immune research

Although the assumption that sleep is important for immune functions has existed for a long time, the systematic investigation of the interactions between sleep and the immune system is a relatively new research area. Therefore, several questions remain open for future studies in this field. The mechanisms underlying these complex neuro-immune interactions are still not well understood. It is likely that different parameters act in concert to mediate the impact of sleep on the immune system and vice versa. Importantly, studies in humans relating sleep-induced changes observed in several immune parameters, like immune cell numbers, to actual infection risk and outcome are lacking so far. The causal involvement of sleep-related immune changes for the development or amplification of chronic inflammatory or infectious diseases described in section II*D* also needs further investigation. In the clinical setting we should take the opportunity to monitor sleep in conditions of acute and chronic immune activation and to assess the impact of specific immunomodulatory treatments to gain further insights into sleep-immune relationships. The effects of sleep on some specific immune aspects, such as autoimmunity (see, e.g., Ref. 419), allergy, tumor immunity, and graft rejection, are under-studied. Tregs, which dampen the immune response in an antigen-specific manner, are dysfunctional in these conditions, and studying the effects of sleep on these cells offers high-yield research opportunities. Large interindividual differences exist with regard to sleep need (the optimal sleep duration for an individual), preferred timing of sleep (the optimal time of day to sleep), and susceptibility to the physiological (and perceptual) consequences of sleep loss, but not much is known about how these differences relate to immune functions. In addition, further research is needed to understand how sleep can be improved to foster its immunosupportive and inflammation-regulatory functions. Nevertheless, substantial knowledge accumulated over the last decades, showing that the sleep-immune crosstalk is a prime example of the research within the overarching field of psychoneuroimmunology. Understanding these interactions in more detail will therefore also lead to a better understanding of basic interactions between the CNS and the immune system, which play an essential role not only in normal physiology, but also in many pathological conditions.

## GRANTS

This work was supported by Deutsche Forschungsgemeinschaft Grants BE 6319/1–1 and TR/SFB 654 C6; National Heart, Lung, and Blood Institute Grant HL136310; and National Institute of Neurological Disorders and Stroke Grant NS091177.

## DISCLOSURES

No conflicts of interest, financial or otherwise, are declared by the authors.
